# Comprehensive analysis of co-expressed genes with TDP-43: prognostic and therapeutic potential in lung adenocarcinoma

**DOI:** 10.1007/s00432-023-05554-9

**Published:** 2024-01-28

**Authors:** Hao Zhang, Juntang Lin, Badrul Hisham Yahaya

**Affiliations:** 1 Lung Stem Cell and Gene Therapy Group (LSCGT), Department of Biomedical Sciences, Advanced Medical and Dental Institute (IPPT), Universiti Sains Malaysia, SAINS@Bertam, 13200 Kepala Batas, Penang, Malaysia; 2 Henan Joint International Research Laboratory of Stem Cell Medicine, School of Medical Engineering, Xinxiang Medical University, Xinxiang, 453003 China

**Keywords:** Lung adenocarcinoma, TDP-43, Co-expressed gene, Prognosis, Tumor microenvironment, Immunotherapy, Drug sensitivity, Bioinformatics analysis

## Abstract

**Background:**

Transactivating DNA-binding protein 43 (TDP-43) is intimately associated with tumorigenesis and progression by regulating mRNA splicing, transport, stability, and non-coding RNA molecules. The exact role of TDP-43 in lung adenocarcinoma (LUAD) has not yet been fully elucidated, despite extensive research on its function in various cancer types. An imperative aspect of comprehending the underlying biological characteristics associated with TDP-43 involves investigating the genes that are co-expressed with this protein. This study assesses the prognostic significance of these co-expressed genes in LUAD and subsequently explores potential therapeutic strategies based on these findings.

**Methods:**

Transcriptomic and clinical data pertaining to LUAD were retrieved from open-access databases to establish an association between mRNA expression profiles and the presence of TDP-43. A risk-prognosis model was developed to compare patient survival rates across various groups, and its accuracy was also assessed. Additionally, differences in tumor stemness, mutational profiles, tumor microenvironment (TME) characteristics, immune checkpoints, and immune cell infiltration were analyzed in the different groups. Moreover, the study entailed predicting the potential response to immunotherapy as well as the sensitivity to commonly employed chemotherapeutic agents and targeted drugs for each distinct group.

**Results:**

The TDP-43 Co-expressed Gene Risk Score (TCGRS) model was constructed utilizing four genes: Kinesin Family Member 20A (*KIF20A*), WD Repeat Domain 4 (*WDR4*), Proline Rich 11 (*PRR11*), and Glia Maturation Factor Gamma (*GMFG*). The value of this model in predicting LUAD patient survival is effectively illustrated by both the Kaplan–Meier (K–M) survival curve and the area under the receiver operating characteristic curve (AUC-ROC). The Gene Set Enrichment Analysis (GSEA) revealed that the high TCGRS group was primarily enriched in biological pathways and functions linked to DNA replication and cell cycle; the low TCGRS group showed primary enrichment in immune-related pathways and functions. The high and low TCGRS groups showed differences in tumor stemness, mutational burden, TME, immune infiltration level, and immune checkpoints. The predictions analysis of immunotherapy indicates that the Tumor Immune Dysfunction and Exclusion (TIDE) score (*p* < 0.001) and non-response rate (74% vs. 51%, *p* < 0.001) in the high TCGRS group are higher than those in the low TCGRS group. The Immune Phenotype Score (IPS) in the high TCGRS group is lower than in the low TCGRS group (*p* < 0.001). The drug sensitivity analysis revealed that the half-maximal inhibitory concentration (IC50) values for cisplatin, docetaxel, doxorubicin, etoposide, gemcitabine, paclitaxel, vincristine, erlotinib, and gefitinib (all *p* < 0.01) in the high TCGRS group are lower than those in the low TCGRS group.

**Conclusions:**

The TCGRS derived from the model exhibits a reliable biomarker for evaluating both prognosis and treatment effectiveness among patients with LUAD. This study is anticipated to offer valuable insights into developing effective treatment strategies for this patient population. It is believed that this study is anticipated to contribute significantly to clinical diagnostics, the development of therapeutic drugs, and the enhancement of patient care.

## Introduction

Lung cancer is recognized as a prominent contributor to cancer-associated deaths, and it stands among the most prevalent malignancies globally (Siegel et al. 2021). Future projections indicate that the United States will encounter approximately 1.9 million new cancer cases and 610,000 deaths linked to cancer by 2023 (Siegel et al. 2023). Among these cases, lung cancer is projected to account for approximately 25% of newly diagnosed cancer cases, carrying a mortality rate of around 42% (Siegel et al. 2023). Consequently, timely diagnosis and development of treatment strategies for lung cancer are crucial.

Among the various types of lung cancer, non-small cell lung cancer (NSCLC) emerges as the most prevalent form (Herbst et al. 2018). Within the realm of NSCLC, the specific subtype identified as lung adenocarcinoma (LUAD) exhibits the highest prevalence accounting for around 50% of all reported lung cancer cases (Bray et al. 2018). Surgery is the preferred treatment for LUAD; however, some patients experience postoperative recurrence and metastasis, resulting in suboptimal treatment outcomes. Additionally, identifying patients with early-stage LUAD can be challenging due to factors such as ambiguous symptoms. By the time the disease progresses to an advanced stage, the optimal window for surgical intervention may have already passed. Therefore, surgical intervention for LUAD has certain limitations. In addition to surgery, treatment options for LUAD include chemotherapy, molecular targeting, and immunotherapy. Approximately 30% of LUAD cases possess molecular targets that can be addressed with targeted drugs (Peng et al. 2023). For patients with advanced LUAD who are not eligible for molecularly targeted treatment, immune checkpoint inhibitors (ICIs) are administered, resulting in a substantial increase in 5-year survival rates from under 5% during chemotherapy to around 30% (Reck et al. 2021). As a result, immunotherapy has been recognized as a viable approach for managing various malignancies, including LUAD. It represents the most promising treatment modality for improving the survival rate of cancer patients (Riley et al. 2019). Despite significant advancements in targeted therapies and immunotherapy, LUAD patients continue to exhibit poor overall survival (OS) rates (Denisenko et al. 2018). Based on literature sources, the relative survival rate of lung cancer over a span of five years is estimated to be around 22% (Siegel et al. 2022). It is crucial to note that only a subset of patients, approximately 40%, exhibit a positive response to ICIs treatment, with even fewer patients achieving long-term remission. This, combined with immune-related adverse effects and issues of primary or secondary resistance, diminishes the efficacy of immunotherapy (Peng et al. 2023; Miller and Hanna 2021; Herbst et al. 2020). As a result, identifying new treatment targets or biomarkers for LUAD is crucial to understand the underlying processes that promote the onset and progression of LUAD.

Currently, a careful pathological examination stands as the primary determinant in the clinical decision-making process. Conventional clinical models rely on indicators such as tumor lymph node metastasis (TNM), the staging system, and microvascular invasion to predict the prognosis of LUAD individuals. However, due to the inherent heterogeneity of LUAD, these models prove inadequate, as they merely offer insights into the anatomical location of the lesion and the malignancy level, while neglecting the underlying molecular mechanisms driving the tumor. Consequently, it becomes imperative to develop comprehensive models that cater to the diverse requirements of LUAD treatment protocols. Such models should adequately evaluate therapy effectiveness and accurately predict patient outcomes, facilitating advancement in biological therapeutics for tumors, the development of novel therapeutic strategies for LUAD, and, ultimately, the enhancement of patient outcomes.

Transactivating DNA-binding protein 43 (TDP-43), also called transactivating DNA-binding protein (TARDBP), is a 43 kDa protein capable of binding to DNA and RNA molecules. The *TARDBP* gene encodes this protein (Nonaka and Hasegawa 2018; Chhangani et al. 2021; Klim et al. 2021; Lye and Chen 2022). TDP-43, belonging to the heterogeneous nuclear ribonucleoprotein (hnRNP) family, exhibits a high level of conservation and expression abundance while exerting multiple functions. Comprising 414 amino acid residues, the protein possesses an N-terminal domain (NTD) and two RNA recognition motifs (RRM1 and RRM2) responsible for the specific recognition of nucleic acids containing TG/UG repeats. Additionally, a complex domain is located at the C-terminus (LCD) (Nonaka and Hasegawa 2018; Chhangani et al. 2021; Klim et al. 2021; Lye and Chen 2022). The NTD of TDP-43 demonstrates self-oligomerization and facilitates RNA recruitment for splicing (Jiang et al. 2017). Both a nuclear export signal and a nuclear localization signal are present on TDP-43, which enables it to serve as a link between the nucleus and cytoplasm (Winton et al. 2008). Furthermore, the LCD consists of disordered glycine-rich regions crucial for protein–protein interactions, including hnRNP binding (Buratti et al. 2005). TDP-43 plays essential roles in multiple physiological processes, such as alternative splicing, RNA transcription, and mRNA stability regulation, based on their structural components and corresponding functions (Ma et al. 2021a). Extensive investigations have explored the involvement of TDP-43 in neurodegenerative disorders (de Boer et al. 2020; Suk and Rousseaux 2020; Carlos and Josephs 2022). In recent years, TDP-43 has emerged as a vital factor in the advancement of malignant tumors, including breast cancer (Ke et al. 2018; Guo et al. 2022), lung cancer (Yang et al. 2020; Guo et al. 2015; Chen et al. 2018), melanoma (Zeng et al. 2017), liver cancer (Liu et al. 2022), and ovarian cancer (He et al. 2023). Studies have demonstrated that TDP-43 interacts with Fas ligand mRNA in lung cancer, enhancing the stability of the Fas ligand mRNA, promoting apoptosis, and ultimately impeding lung cancer growth (Yang et al. 2020). According to in vitro studies, TDP-43 regulates the expression of the metastasis-associated lung adenocarcinoma transcript-1, which affects NSCLC cell proliferation, migration, and infiltration (Guo et al. 2015). Moreover, TDP-43 plays a dual role in lung cancer; it promotes cell migration by regulating mir-423-3p while simultaneously inhibiting lung cancer development by regulating mir-500a-3p (Chen et al. 2018). These studies suggest that TDP-43 likely participates in the diverse mechanisms underlying lung cancer development.

There remains a significant knowledge gap regarding the specific contribution of TDP-43 to lung cancer, necessitating further research in this area. Previous investigations have primarily focused on the role of a single gene, whereas carcinogenesis often arises from complex interactions among multiple genes. To gain new insights and develop effective treatment strategies, a comprehensive investigation into the prognostic significance of genes co-expressed with TDP-43 in LUAD and their relationship with the tumor microenvironment (TME) is necessary. Moreover, it is crucial to identify patients who exhibit responsiveness to immune-based therapies, targeted therapies, and chemotherapy drugs. Notably, there needs to be more research centered on the bioinformatic analysis of gene signatures co-expressed with TDP-43 in LUAD. Therefore, conducting such studies will be instrumental in advancing our comprehension of the intricate molecular mechanisms underlying LUAD and paving the way for developing new therapeutic strategies.

In this current investigation, publicly available data were retrieved to analyze genes co-expressed with TDP-43, with the aim of constructing a risk score model to predict the prognosis of patients diagnosed with LUAD. The model was subsequently validated using three independent cohorts. Furthermore, additional investigations were conducted to explore the characteristics of the model regarding tumor stemness, tumor mutational burden (TMB), TME, and immune responses. The study also predicted the therapeutic potential of the model. These findings offer novel insights into the treatment strategies and prognostic assessment of LUAD.

## Materials and methods

### Sources of research information

In the current study, The Cancer Genome Atlas (TCGA) database (https://portal.gdc.cancer.gov/) was searched (Tomczak et al. 2015) to gather clinical information, mRNA expression data, and somatic mutation data for both normal tissues (*n* = 59) and LUAD tissues (*n* = 535). TMB was determined by analyzing the somatic mutation data. The primary dataset analyzed in this study was The Cancer Genome Atlas Lung Adenocarcinoma (TCGA-LUAD), and supplementary LUAD data were sourced from the Gene Expression Omnibus (GEO) database (http://www.ncbi.nlm.nih.gov/geo/), including GSE72094 (*n* = 442) (Schabath et al. 2016), GSE68465 (*n* = 443) (Shedden et al. 2008), and GSE41271 (*n* = 181) (Sato et al. 2013). Copy number variations (CNVs) data for the TCGA-LUAD cohort were retrieved from the UCSC Xena database (https://xena.ucsc.edu/) (Goldman et al. 2020). Furthermore, the CEO database was searched to retrieve single-cell RNA-seq data for 42 patients with NSCLC (GSE148071) (Wu et al. 2021a).

### Screening of genes and CNVs analysis

To identify genes co-expressed with TDP-43, the "limma" package of R (3.54.0) was employed to assess the TCGA-LUAD mRNA expression data. Genes were categorized as positively associated (correlation coefficient > 0.3 and *p* < 0.05) or negatively associated (correlation coefficient < −0.3 and *p* < 0.05). These TDP-43 co-expressed genes were further analyzed in three additional LUAD datasets. The R package "VennDiagram" (1.7.3) was employed to visualize overlapping genes among the four datasets. Differentially expressed genes (DEGs) in TCGA-LUAD were assessed using the "limma" package of R (3.54.0), with a selection threshold of an absolute value of log2 fold change (|log2FC|) > 1.0 and a false discovery rate (FDR) < 0.05. Prognostic-related genes (*p* < 0.05) were identified utilizing the R package "survival" (3.4–0). The R package "Venn" (1.11) assisted in visualizing the genes with differential expression and prognostic-related characteristics. CNVs for the characteristic genes were presented in CNVs frequency plots. The distribution of these characteristic genes across different chromosomes was visualized using the R package "RCircos" (1.2.2).

### Construction and validation of the prognostic model, independent prognostic risk factor analysis, and nomogram establishment

The "least absolute shrinkage and selection operator" (LASSO) regression analysis was carried out using the R package "glmnet" (4.1–6) (Friedman et al. 2010), with ten-fold cross-validation being used for gene selection and model development. Risk scores were computed based on the formulas below:

$${\text{TDP}}-43\mathrm{ Co}-\text{expressed Gene Risk Score }({\text{TCGRS}})\hspace{0.17em}=\hspace{0.17em}{\sum\limits_{i=1}^{n}}Coef \left(i\right)\times Exp (i)$$

The regression coefficient (Coef ($i$) and gene expression level Exp ($i$) were used in the current investigation. Patients were categorized into high and low TCGRS groups based on the cutoff value derived from the receiver operating characteristic (ROC) curve. The high TCGRS group was considered high risk, while the low TCGRS was considered low risk. The R package "pheatmap" (1.0.12) was employed to generate survival scatter plots, heat maps, and risk curve plots. To analyze the overall survival (OS) rates and create survival curves for patients in the high and low TCGRS groups, the R packages "survival” (3.4–0) and “survminer” (0.4.9) were utilized. The R package "timeROC" (0.4) was used to plot 1, 3, and 5-year ROC curves and calculated the corresponding area under the curve (AUC) values. The GSE72094, GSE68465, and GSE41271 datasets were employed for further model validation. Cox regression analysis, performed using the R package "survival" (3.4–0), assessed TCGRS as an independent prognostic factor for LUAD. A nomogram based on TCGA-LUAD was established using a combination of R packages, including "survival" (3.4–0), "survminer" (0.4.9), "timeROC" (0.4), "rms" (6.3–0), and "regplot" (1.1). The clinical utility of the nomogram was evaluated using 1-, 3-, and 5-year clinical decision analysis (DCA) curves generated by the R package "ggDCA" (1.2).

### Meta-analysis of genes used in model construction

The "Lung Cancer Explorer" (LCE) visualization platform (https://lce.biohpc.swmed.edu/lungcancer/) (Cai et al. 2019) was employed to perform a meta-analysis of the model genes, presenting the results as forest plots. For gene expression comparisons, the standardized mean difference (SMD) was used as the analytical statistic, while the hazard ratio (HR) served as the analytical statistic for survival comparisons. A random-effects model was implemented for statistical testing, with the 95% confidence interval (CI) providing the interval estimate for each statistic.

### Gene set enrichment analysis (GSEA)

GSEA is a computational technique that evaluates predefined gene subsets to determine whether gene sets in various risk groups are differentially enriched across certain phenotypic categories. This powerful approach can enable the identification of common biological pathways (Subramanian et al. 2005). The R packages "clusterProfiler" (4.6.0), "enrichplot" (1.18.3), and "org.Hs.eg.db" (3.16.0) (Yu et al. 2012) were employed to perform GSEA analysis on the different TCGRS groups. This research facilitated the determination of potential biological activities and pathways linked to the groups using Gene Ontology (GO) and Kyoto Encyclopaedia of Genes and Genomes (KEGG) gene sets as references.

### Analysis of the correlation between the TCGRS and stemness characteristics

The high expression of biomarkers correlated with tumor stem cells is strongly linked to tumor proliferation, cancer recurrence, and drug resistance (Luo and Vögeli 2020). Stemness scores, which characterize the similarity between tumor cells and stem cells, can be derived using the "one-class logistic regression" (OCLR) algorithm (Malta et al. 2018). The stemness score calculated from mRNA expression data is referred to as "mRNAss,” while the score calculated from methylation data is denoted as "mDNAss.” To evaluate and visualize the differences in "mRNAss" and "mDNAss" between the high and low TCGRS groups, the R package "ggpubr" (0.5.0) was employed.

### TMB analysis

The top 20 mutated genes in the high- and low- TCGRS groups were displayed, and the TMB was calculated using the R package "maftools" (2.14.0). The differences in TMB between the high- and low- TCGRS groups were assessed and visualized using the R package "ggpubr" (0.5.0).

### TME, immune cell infiltration, and immune checkpoint analyses

The "ESTIMATE" method was used to estimate the proportion of immune and stromal cells in the TME of tumor samples (Yoshihara et al. 2013). The R package "estimate" (1.0.13) was used to determine stromal scores, immune scores, and tumor purity based on TCGA-LUAD data. The "ESTIMATE" score is obtained by the sum of the stromal and immune scores. The "CIBERSORT" algorithm, a deconvolution technique, can predict the relative abundance of immune cell populations (Newman et al. 2015). To estimate the abundance of 22 tumor-infiltrating immune cells in TCGA-LUAD samples, the R package "CIBERSORT" (1.03) was utilized. Additionally, the expression levels of multiple immune checkpoints (Danilova et al. 2019) were compared to analyze potential differences between high and low TCGRS groups.

### Anticipation of the immune therapeutic reaction and drug sensitivity

The assessment of immunotherapy efficacy in patients often involves the calculation of the "Tumor Immune Dysfunction and Exclusion" (TIDE) score, which is obtained from the TIDE website (http://tide.dfci.harvard.edu/), and the "Immune Phenotype Score" (IPS), acquired from the "Cancer Immune Atlas" website (https://tcia.at/). A lower TIDE score and a higher IPS indicate favorable immunotherapy outcomes. The R package "pRRophetic" (0.5.1) was employed to predict the half-maximal inhibitory concentration (IC50) value of the target drug utilizing the "Cancer Drug Sensitivity Genomics" database (https://www.cancerrxgene.org/). A higher IC50 value suggests lower drug sensitivity.

### Single-cell analysis

The "Tumor Immune Single-cell Hub" (TISCH) database (http://tisch.comp-genomics.org/) comprises high-quality transcriptome data with single-cell level cell type annotations (Sun et al. 2021). The "GSE148071" dataset was retrieved from the database, and "Uniform Manifold Approximation and Projection" (UMAP) plots were utilized to display the expression and distribution of model genes in different immune cell types.

### Analysis of protein expression levels

The protein expression of the model genes was analyzed utilizing the "Human Protein Atlas" (HPA) database (https://www.proteinatlas.org/), which provides free access to immunohistochemical slides. A positive immune response can be determined when nuclear staining is observed in tumor cells.

### Statistical analysis

The Wilcoxon test was conducted to evaluate variations between the two groups, with the exception of the evaluation of immunotherapy (categorical data), which was compared by means of the chi-square test. The Kaplan–Meier (K–M) method was employed to estimate the survival rates for high and low TCGRS groups. Numerical variables or combinations of categorical and numerical variables were analyzed utilizing univariate and multivariate Cox regression. TDP-43 co-expressed correlated genes were analyzed using Pearson’s method, while correlations between tumor stemness score, TMB, TME, immune cell infiltration, TIDE score, and TCGRS were analyzed using Spearman’s method. Statistical significance was defined as *p* < 0.05.

## Results

### Workflow and clinical characteristics of the research data

**Fig. 1:**
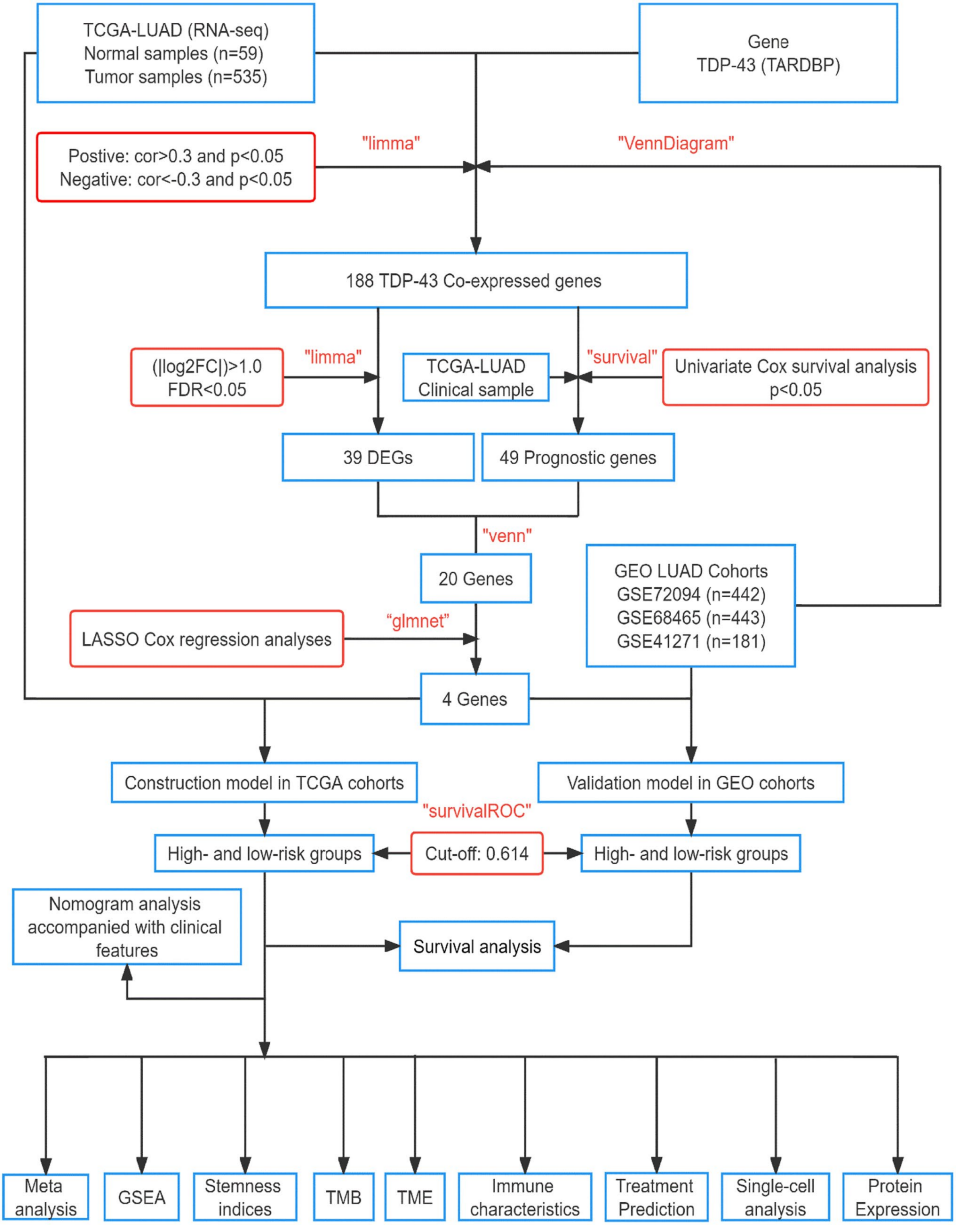
The workflow and approach of this study

The study workflow is depicted in Fig. 1. TCGA-LUAD dataset comprises clinical data from 522 patients with LUAD (13 patients with unknown survival time were excluded). Table 1 presents the clinical information for four LUAD datasets.

**Table 1:** Clinical baseline characteristics of LUAD patients involved in this study

Characteristics	TCGA-LUAD	GSE72094	GSE68465	GSE41271
Cases	522	442	443	181
Age (years), Median	66	70	65	63
< 65	223 (42.72%)	115 (26.02%)	214 (48.31%)	99 (54.70%)
≥ 65	280 (53.64%)	306 (69.23%)	229 (51.69%)	82 (45.30%)
Unknown	19 (3.64%)	21 (4.75%)	0	0
Gender				
Female	280 (53.64%)	240 (54.30%)	220 (49.66%)	90 (49.72%)
Male	242 (46.36%)	202 (45.70%)	223 (50.34%)	91 (50.28%)
Clinical Stage			NA	
I	279 (53.45%)	265 (59.95%)		100 (55.25%)
II	124 (23.76%)	69 (15.61%)		28 (15.47%)
III	85 (16.28%)	63 (14.25%)		49 (27.07%)
IV	26(4.98%)	17 (3.85%)		4 (2.21%)
Unknown	8 (1.53%)	28 (6.34%)		0
T Stage		NA		NA
T1	172 (32.95%)		150 (33.86%)	
T2	281 (53.83%)		251 (56.66%)	
T3	47 (9.00%)		28 (6.32%)	
T4	19 (3.64%)		12 (2.71%)	
Unknown	3 (0.58%)		2 (0.45%)	
N Stage		NA		NA
N0	335 (64.18%)		299 (67.49%)	
N1	98 (18.77%)		88 (19.87%)	
N2	75 (14.37%)		53 (11.96%)	
N3	2 (0.38%)		NA	
Unknown	12 (2.30%)		3 (0.68%)	
M Stage		NA	NA	NA
M0	353 (67.62%)			
M1	25 (4.79%)			
Unknown	144 (27.59%)			
Survival time(days), Median	551	824	1410	1178
Unknown	0	44 (9.95%)	1 (0.23%)	0
Survival State				
Alive	355 (68.01%)	298 (67.42%)	207 (46.73%)	112 (61.88%)
Dead	167 (31.99%)	122 (27.60%)	236 (53.27%)	69 (38.12%)
Unknown	0	22 (4.98%)	0	0

*NA* Not available

### Screening genes co-expressed with TDP-43 and prognosis-related genes and CNVs analysis

**Fig. 2:**
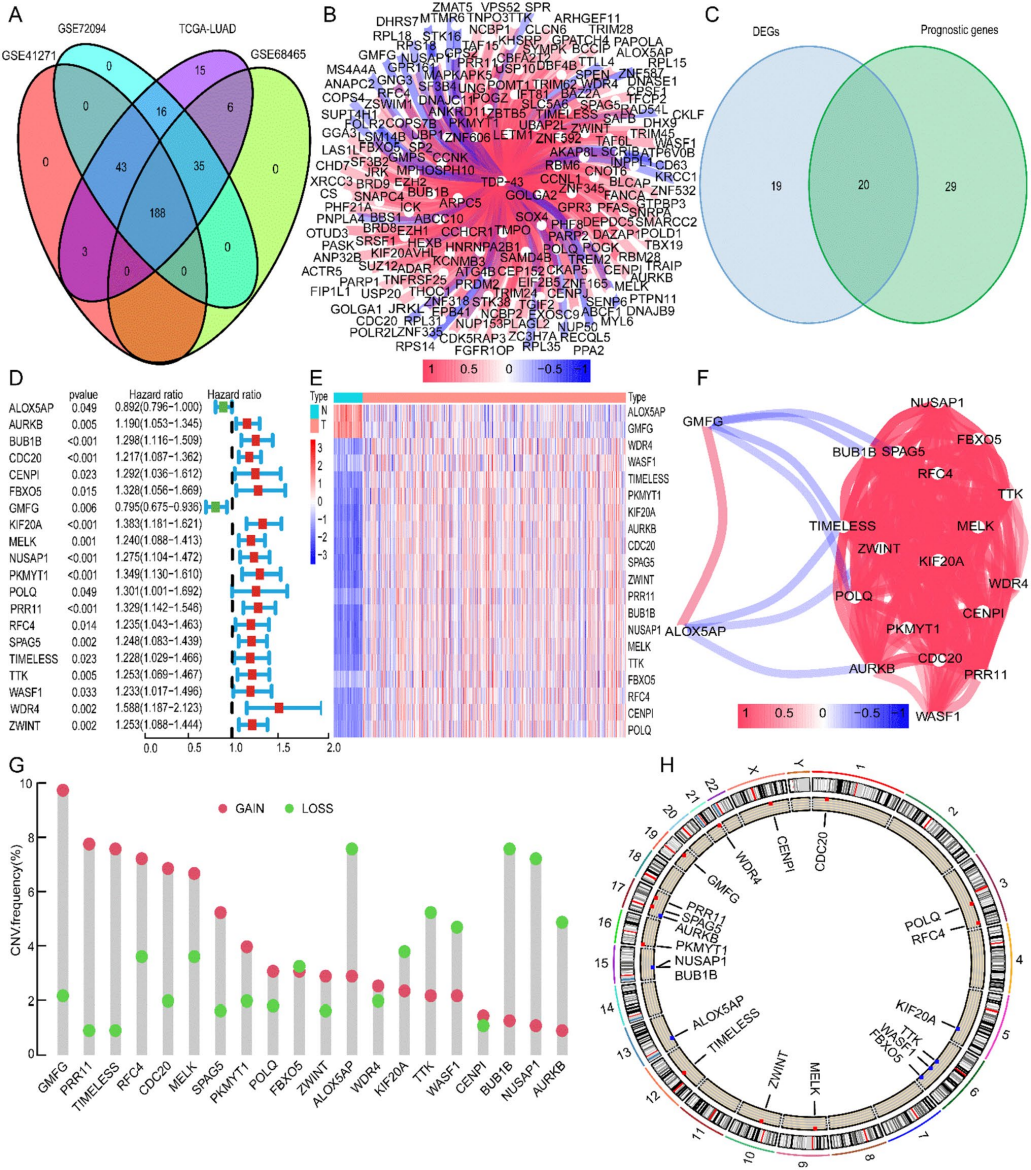
Screening for co-expressed genes with TDP-43 and analysis of CNVs in LUAD. **A** Using data from four LUAD cohorts (TCGA-LUAD, GSE72094, GSE68465, and GSE41271), we identified 188 genes co-expressed with TDP-43. A positive correlation is depicted in red, whereas a negative connection is shown in blue. **B** The correlation between TDP-43 and 188 genes. **C** Utilizing the TCGA-LUAD cohort, 20 genes associated with differential expression and prognosis were identified. **D** Findings from a survival study using univariate Cox regression on 20 genes. **E** Expression of 20 genes in normal and tumor samples from the TCGA-LUAD cohort. High expression is indicated by red, whereas low expression is indicated by blue. **F** The correlation analysis network diagram of 20 genes. **G** CNVs frequency changes in LUAD for 20 genes. The amplification frequency is shown in red, and the deletion frequency is shown in green. **H** Location on LUAD chromosomes of 20 genes with altered CNVs

In TCGA-LUAD, a total of 306 co-expressed genes with TDP-43 were identified. Further analysis of gene expression using GEO datasets revealed 188 genes that were consistently co-expressed across the four datasets (Figs. 2A and B). Among these 188 genes, 39 were identified as DEGs, and 49 were identified as prognostic-related genes in TCGA-LUAD, resulting in a set of 20 genes that were both differentially expressed and prognostically relevant (Fig. 2C). Cox regression analysis revealed that high expression of 18 genes was associated with poor prognosis, while low expression of two genes was also linked to poor prognosis (Fig. 2D). The expression patterns of these 20 genes in the samples are illustrated in Fig. 2E, and their correlations are depicted in Fig. 2F. Analysis of CNVs revealed that the amplification frequency was higher for 12 genes, while the deletion frequency was higher for eight genes (Fig. 2G). Additionally, Fig. 2H provides the visualization of the chromosomal locations of these 20 genes.

### TCGRS model construction and validation and nomogram establishment

**Fig. 3:**
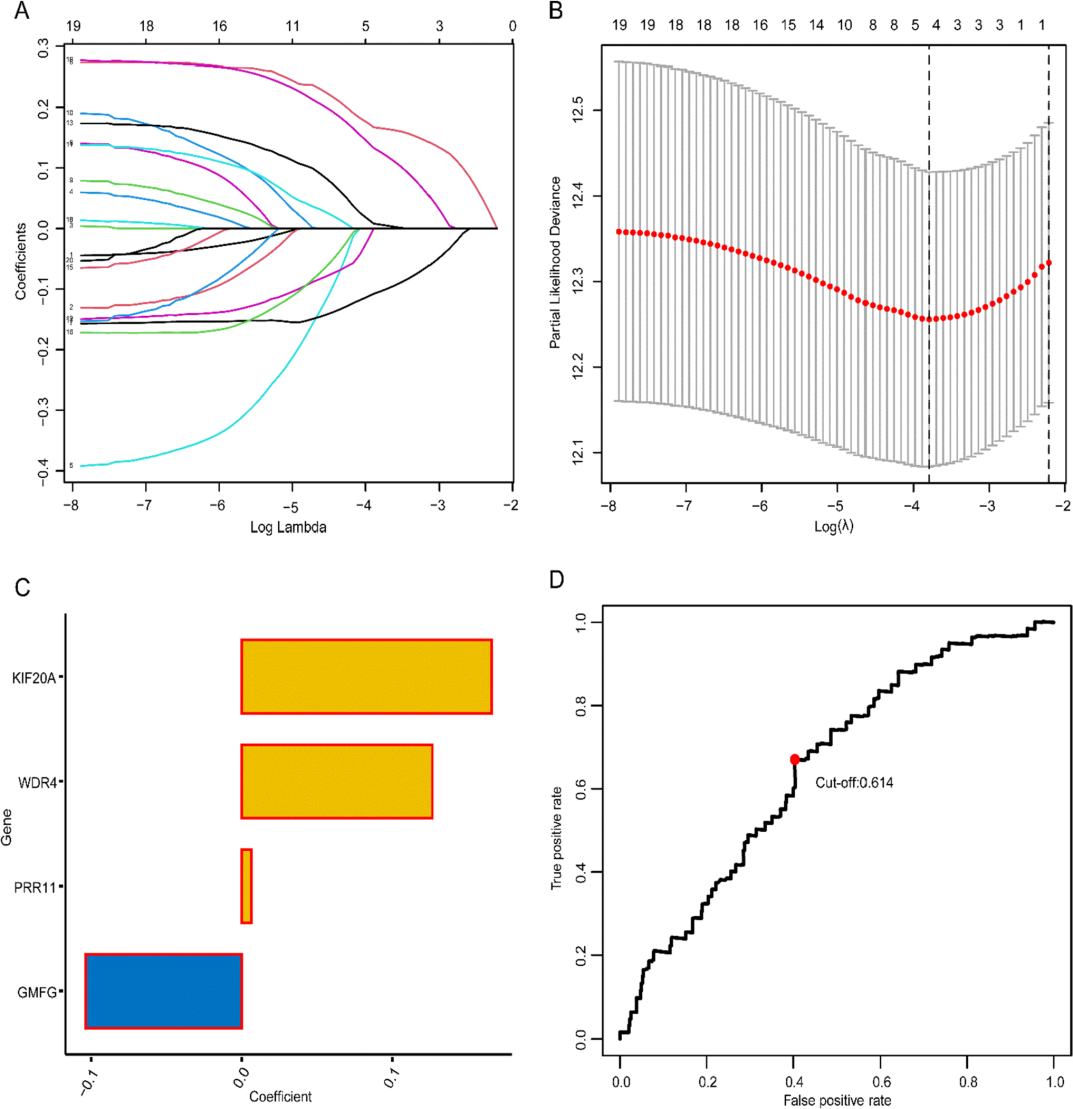
LASSO regression analysis were utilized to construct the TCGRS model. **A** The process of constructing the LASSO regression coefficient route for the model. **B** Cross-validation curves for model construction. **C** Regression coefficients for participating model genes. **D** The model determined the ROC curve to get the best possible cut-off value for TCGRS

Through LASSO regression, a model was constructed from 4 genes selected from the 20 differentially expressed-prognostic genes based on TCGA-LUAD, namely Kinesin Family Member 20A (*KIF20A*), WD Repeat Domain 4 (*WDR4*), Proline Rich 11 (*PRR11*), and Glia Maturation Factor Gamma (*GMFG*), as shown in Fig. 3. The pertinent data and regression coefficients for these model genes are presented in Table 2. The model formula is given below:

**Table 2:** Basic information for constructing model genes

Gene Symbol	Description	TDP-43 Relevance	P-value	Type	Log2FC	FDR	LASSO Coefficient
KIF20A	Kinesin Family Member 20A	0.400313	5.22E-22	Positive	3.308652	3.34E-32	0.165598899115021
WDR4	WD Repeat Domain 4	0.315061	8.57E-14	Positive	1.060900	1.85E-24	0.126214693287426
PRR11	Proline Rich 11	0.330059	4.61E-15	Positive	2.393045	8.62E-24	0.00639771240580875
GMFG	Glia Maturation Factor Gamma	−0.37769	1.39E-19	Negative	−1.326675	2.00E-27	-0.10373143883509

TCGRS = (0.165598899115021) × (*KIF20A* expression levels) + (0.126214693287426) × (*WDR4* expression levels) + (0.00639771240580875) × (*PRR11* expression levels) + (-0.10373143883509) × (*GMFG* expression levels).

**Fig. 4:**
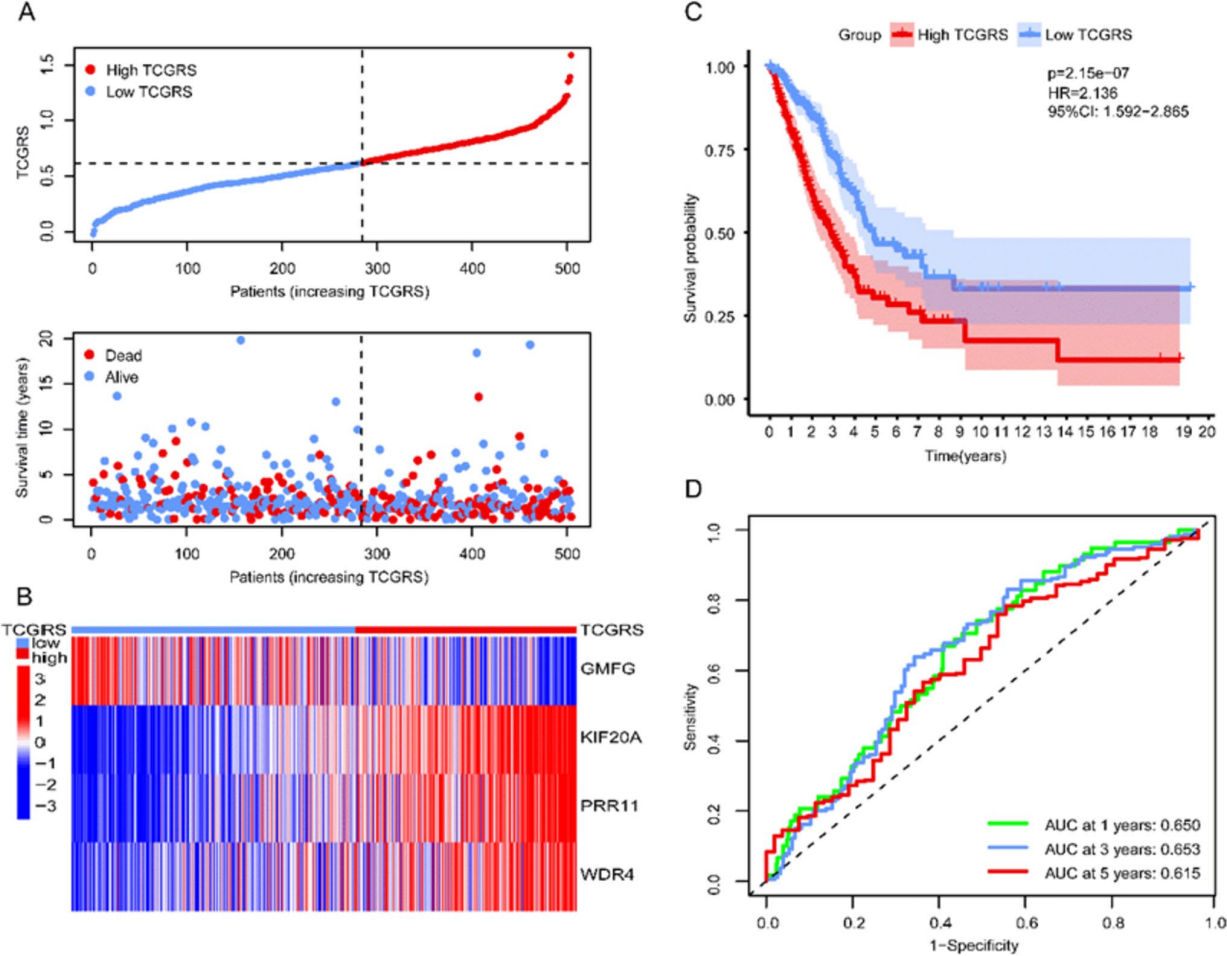
The prognostic value of the TCGRS model was constructed using the TCGA-LUAD cohort. **A** The distribution of TCGRS and survival status among patients in this cohort. **B** Compares model gene expression levels between patients with high and low TCGRS groups. **C** The K–M survival curves for high and low TCGRS groups. **D** The ROC curves for 1-, 3-, and 5-year survival predictions are based on the TCGRS model

**Fig. 5:**
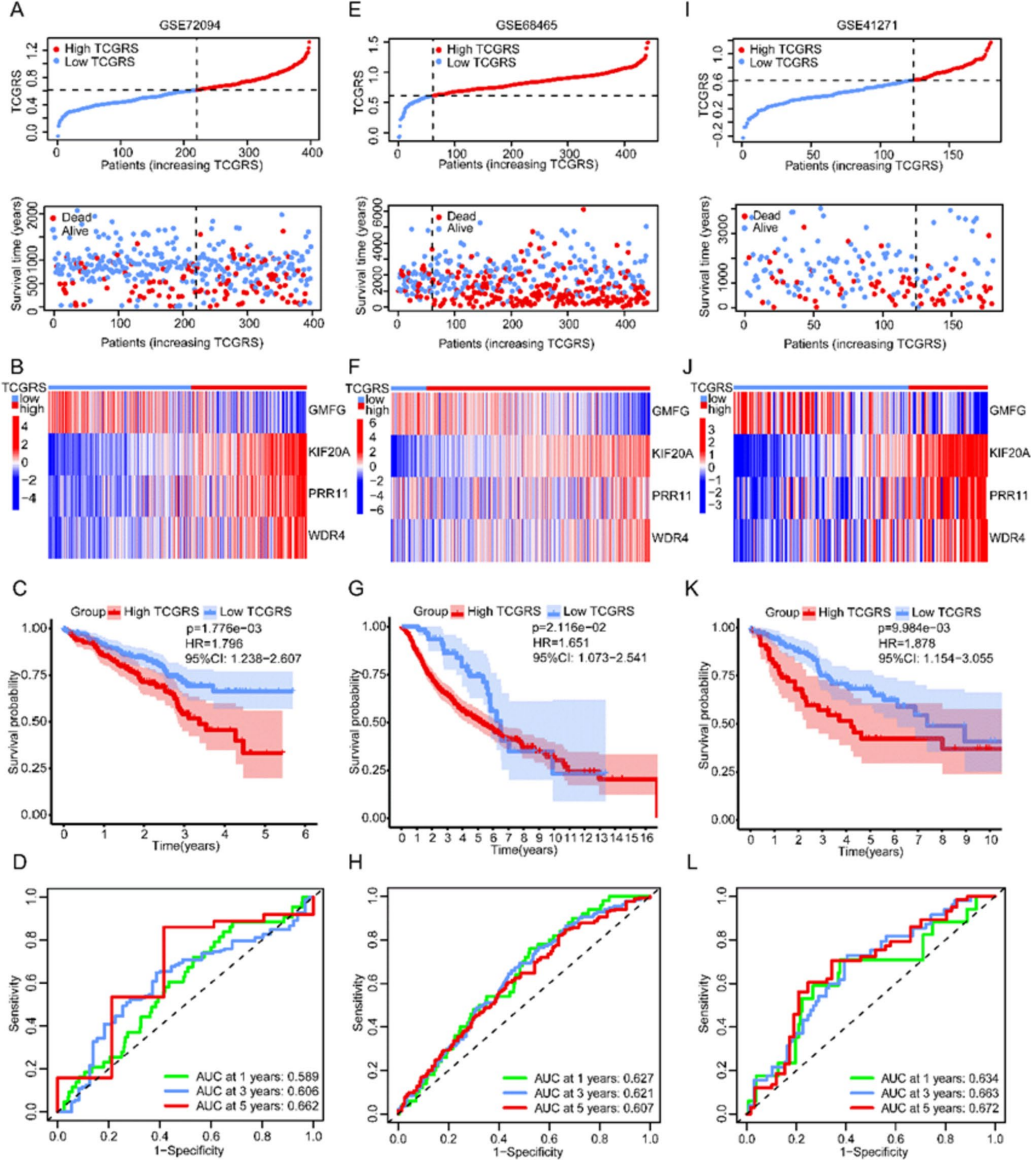
Assessing the prognostic value of the TCGRS model using three validation cohorts. **A** Distribution of TCGRS and survival status in the GSE72094 cohort. **B** Model gene expression in high and low TCGRS groups in the GSE72094 cohort. **C** Survival curve of the GSE72094 cohort. **D** ROC curve of the GSE72094 cohort. **E** Distribution of TCGRS and survival status in the GSE68465 cohort. (F) Model gene expression in high and low TCGRS groups in the GSE68465 cohort. **G** Survival curve of the GSE68465 cohort. **H** ROC curve of the GSE68465 cohort. **I** Distribution of TCGRS and survival status in the GSE41271 cohort. (J) Model gene expression in high and low TCGRS groups in the GSE41271 cohort. (K) Survival curve of the GSE41271 cohort. **L** ROC curve of the GSE41271 cohort

**Fig. 6:**
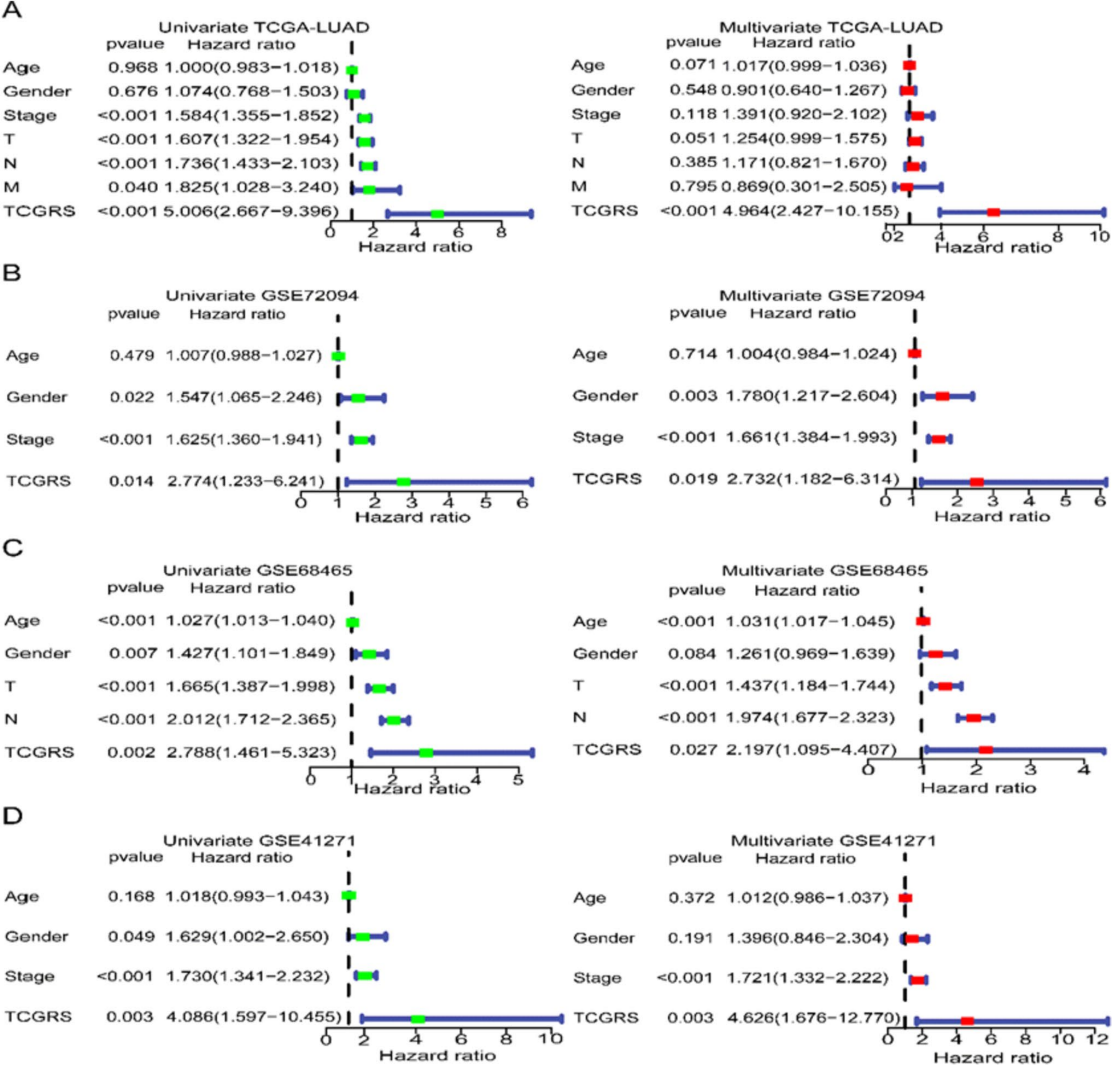
Cox regression analysis of TCGRS in four LUAD cohorts: results from univariate and multivariate analyses. The findings of a univariate Cox regression analysis are displayed on the left, while those of a multivariate analysis are displayed on the right. **A** TCGA-LUAD cohort. **B** GSE72094 cohort. **C** GSE68465 cohort. **D** GSE41271 cohort

**Fig. 7:**
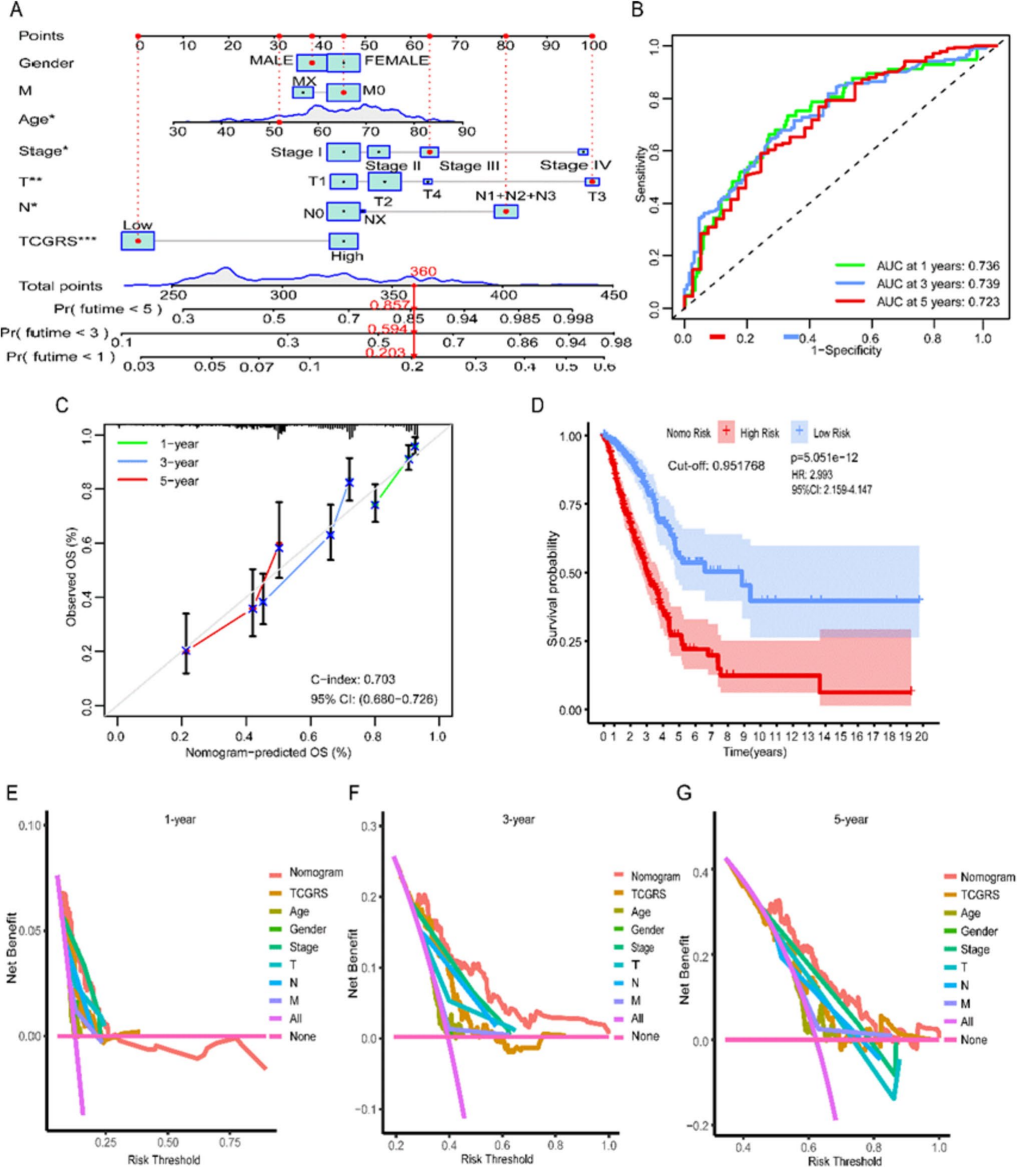
A nomogram for predicting OS in LUAD is constructed using clinical data and TCGRS from the TCGA-LUAD cohort. **A** 1-, 3-, and 5-year OS of LUAD patients were predicted using combined gender, age, clinical stage, T stage, N stage, M stage, and TCGRS constructed nomograms from the TCGA-LUAD cohort. **B** ROC curves reflect the ability of nomograms to assess 1-, 3-, and 5-year survival outcomes in LUAD patients. **C** The calibration curves reflect the accuracy of the nomograms in assessing the 1-, 3-, and 5-year survival outcomes of LUAD patients. **D** The K–M curve compared the survival difference between the high and low risk groups based on the nomogram. **E**–**G** DCA to evaluate the clinical utility of nomograms for predicting 1-, 3-, and 5-year OS of LUAD. * *p* < 0.05, ** *p* < 0.01, *** *p* < 0.001

Using a cut-off value of 0.614, the samples were divided into a high TCGRS group (≥ 0.614) and a low TCGRS group (< 0.614). As TCGRS increased, the number of deaths also increased (Fig. 4A). In the high TCGRS group, *KIF20A*, *WDR4*, and *PRR11* expression increased, while in the low TCGRS group, *GMFG* expression increased (Fig. 4B). The high TCGRS group exhibited a shorter survival duration compared to the low TCGRS group (Fig. 4C). The performance of the model was evaluated using ROC curves at 1, 3, and 5 years, as shown in Fig. 4D. To validate the model, three GEO datasets were utilized, highlighting its robust predictions capability for survival (Fig. 5). Cox regression analysis performed on the four datasets confirmed that TCGRS could serve as an independent prognostic factor (Fig. 6). By combining the clinical parameters of the TCGA-LUAD cohort with TCGRS, a nomogram was developed to improve survival prediction, yielding a reliable tool with a good prediction value (Fig. 7A–D). DCA analysis revealed that while the net benefit of the nomogram was not significant at 1 year OS, it was better than other clinical characteristics at 3 years and 5 years (Fig. 7E–G).

### Meta-analysis of gene expression and survival differences

**Fig. 8:**
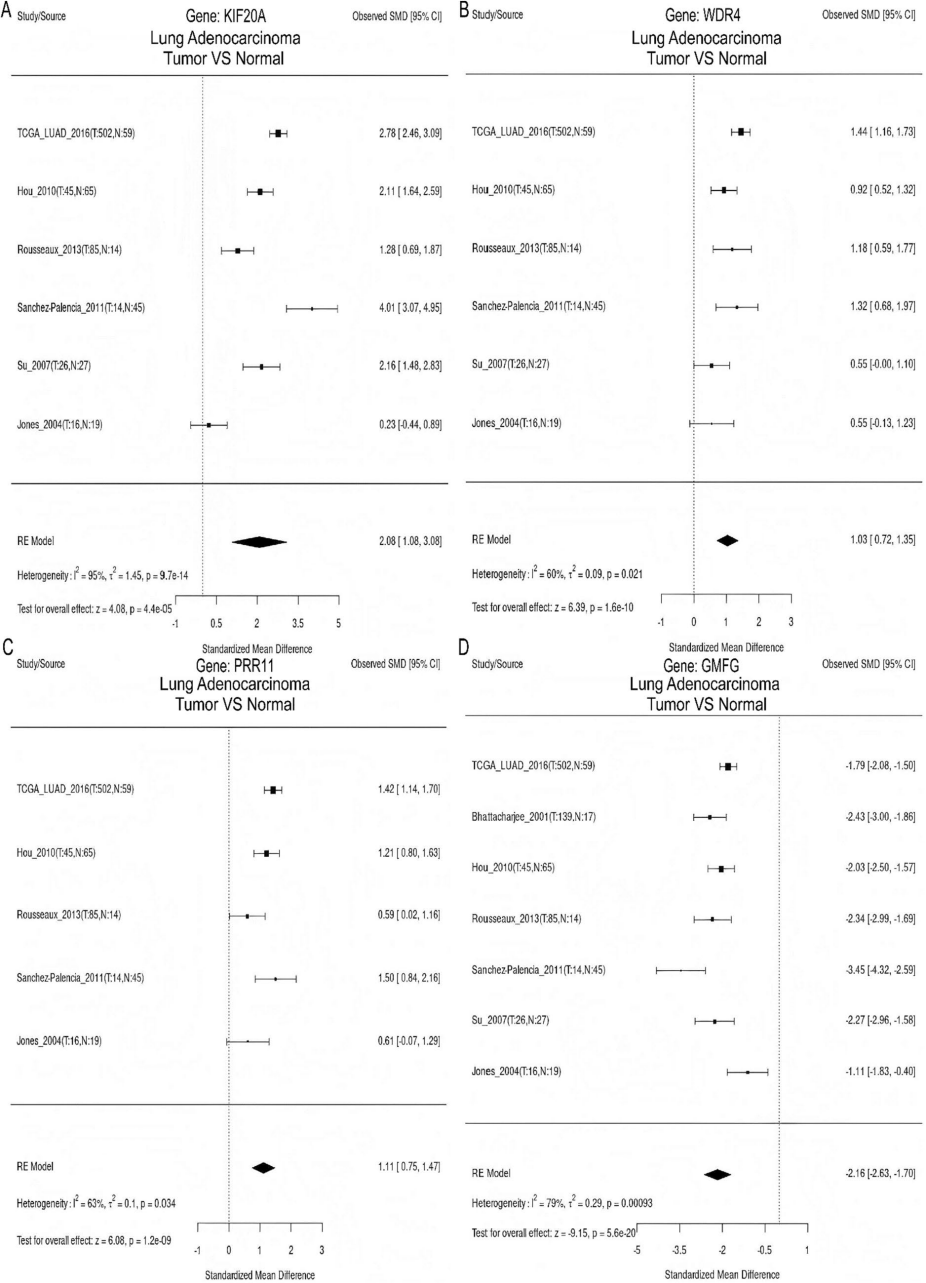
Meta-analysis of model gene expression differences. **A** *KIF20A*. **B** *WDR4*. **C** *PRR11*. **D** *GMFG*

**Fig. 9:**
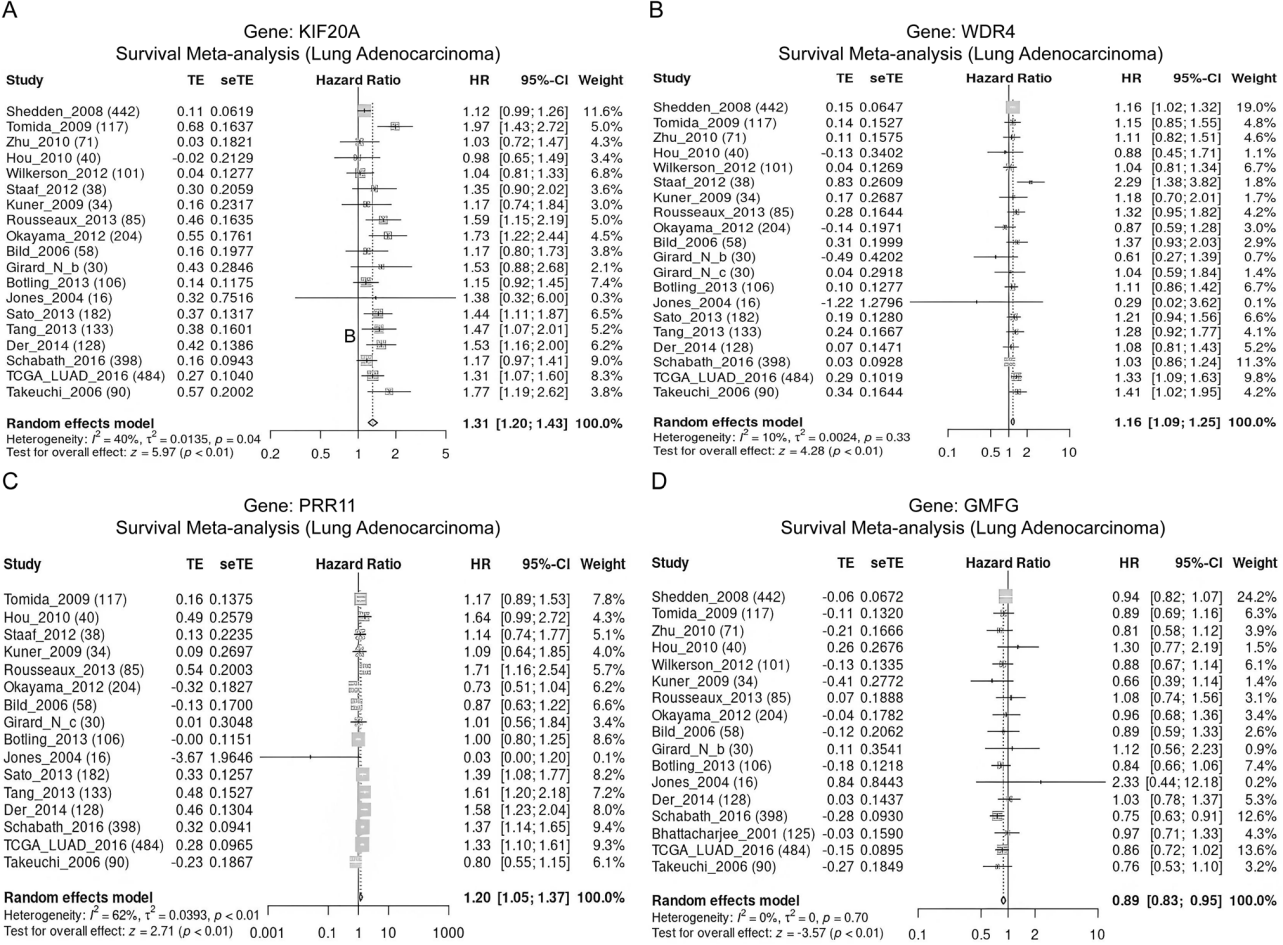
Meta-analysis of survival differences by model genes. **A** *KIF20A*. **B** *WDR4*. **C** *PRR11*. **D** *GMFG*

The meta-analysis demonstrated an upregulation of *KIF20A*, *WDR4,* and *PRR11* expression levels in LUAD tissues, whereas *GMFG* expression level was found to be decreased (Fig. 8). Survival analysis further revealed that high expression of *KIF20A*, *WDR4*, and *PRR11* was considerably correlated with unfavorable prognosis, whereas low expression of *GMFG* was linked to poor prognosis (Fig. 9).

### GSEA

**Fig. 10:**
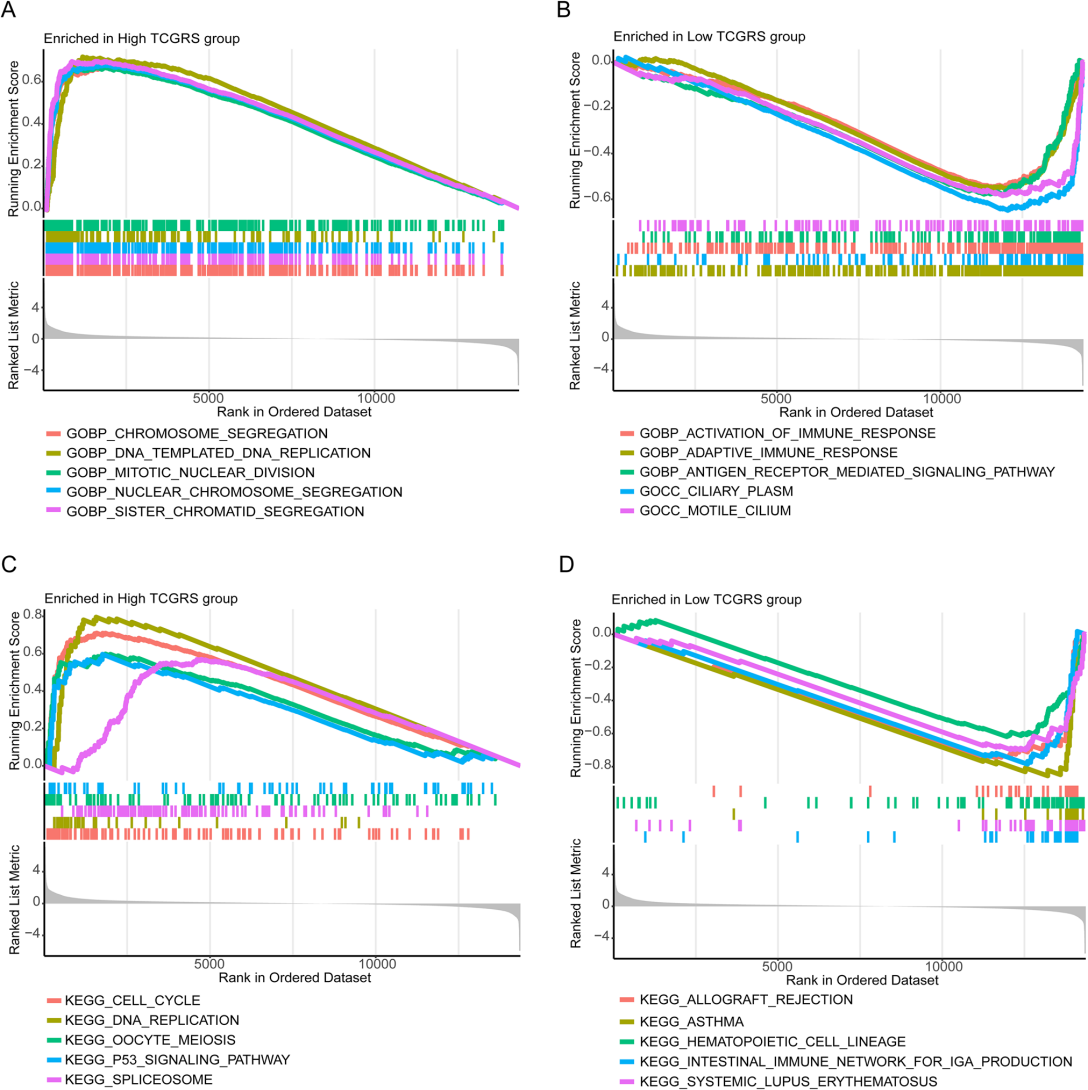
The biological characteristics of different TCGRS groups were assessed by GSEA based on the TCGA-LUAD cohort. **A** GO enrichment analysis of the high TCGRS group. **B** GO enrichment analysis of the low TCGRS group. **C** KEGG enrichment analysis in the high TCGRS group. **D** KEGG enrichment analysis in the low TCGRS group

To study the various biological processes and pathways linked to the high and low TCGRS groups, GSEA was performed using GO and KEGG gene sets as references. The study revealed that the high TCGRS group was primarily enriched in biological pathways and functions linked to DNA replication, cell cycle, and DNA templated. In contrast, the low TCGRS group showed primary enrichment in immune-related pathways and functions (Fig. 10).

### Tumor stem cell signature and TMB analysis

**Fig. 11:**
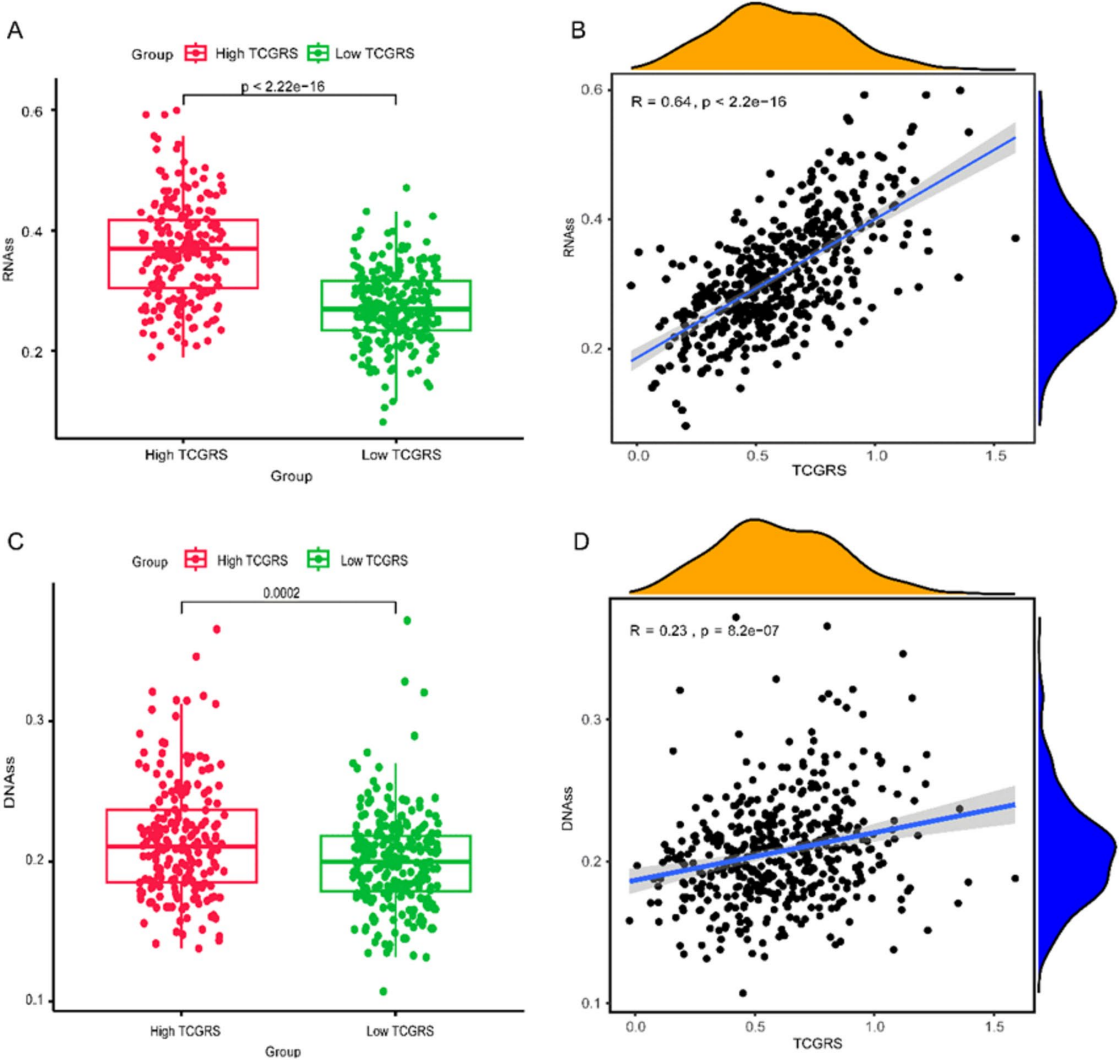
Evaluated differences in RNAss and DNAss between TCGRS groups in the TCGA-LUAD cohort and examined the correlation between TCGRS and stemness scores. **A** Analysis of differences between high and low TCGRS groups in RNAss. **B** Analysis of correlations between TCGRS and RNAss. **C** Analysis of differences between high and low TCGRS groups in DNAss. **D** Analysis of correlations between TCGRS and DNAss

**Fig. 12:**
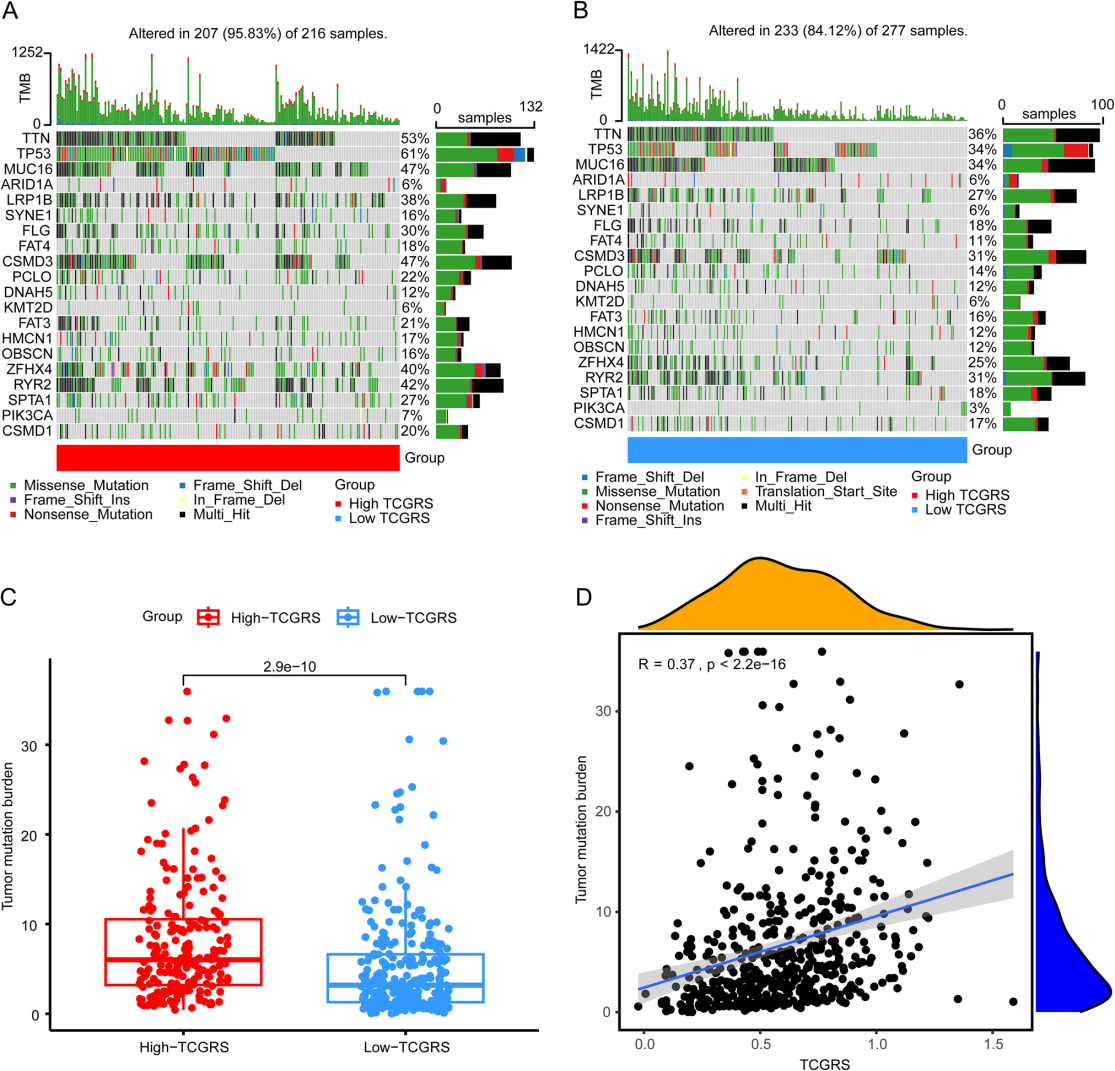
Somatic mutation signatures and TMB analysis in high and low TCGRS groups. **A** Top 20 gene signatures of somatic mutations in high TCGRS group. **B** Top 20 gene signatures of somatic mutations in low TCGRS group. **C** Analysis of differences in TMB between high- and low- TCGRS groups. **D** Analysis of correlations between TCGRS and TMB

The high TCGRS group exhibited elevated levels of both "RNAss" and "DNAss" compared to the low TCGRS group, with a positive correlation between TCGRS and these measures (Fig. 11). Mutation frequencies of the top 20 genes in somatic cells were also higher in the high TCGRS group (Figs. 12A and B). Additionally, TMB analysis showed that TCGRS and TMB had a positive correlation, with the high TCGRS group demonstrating higher levels than the low TCGRS group (Figs. 12C and D).

### Immunological characterization analysis

**Fig. 13:**
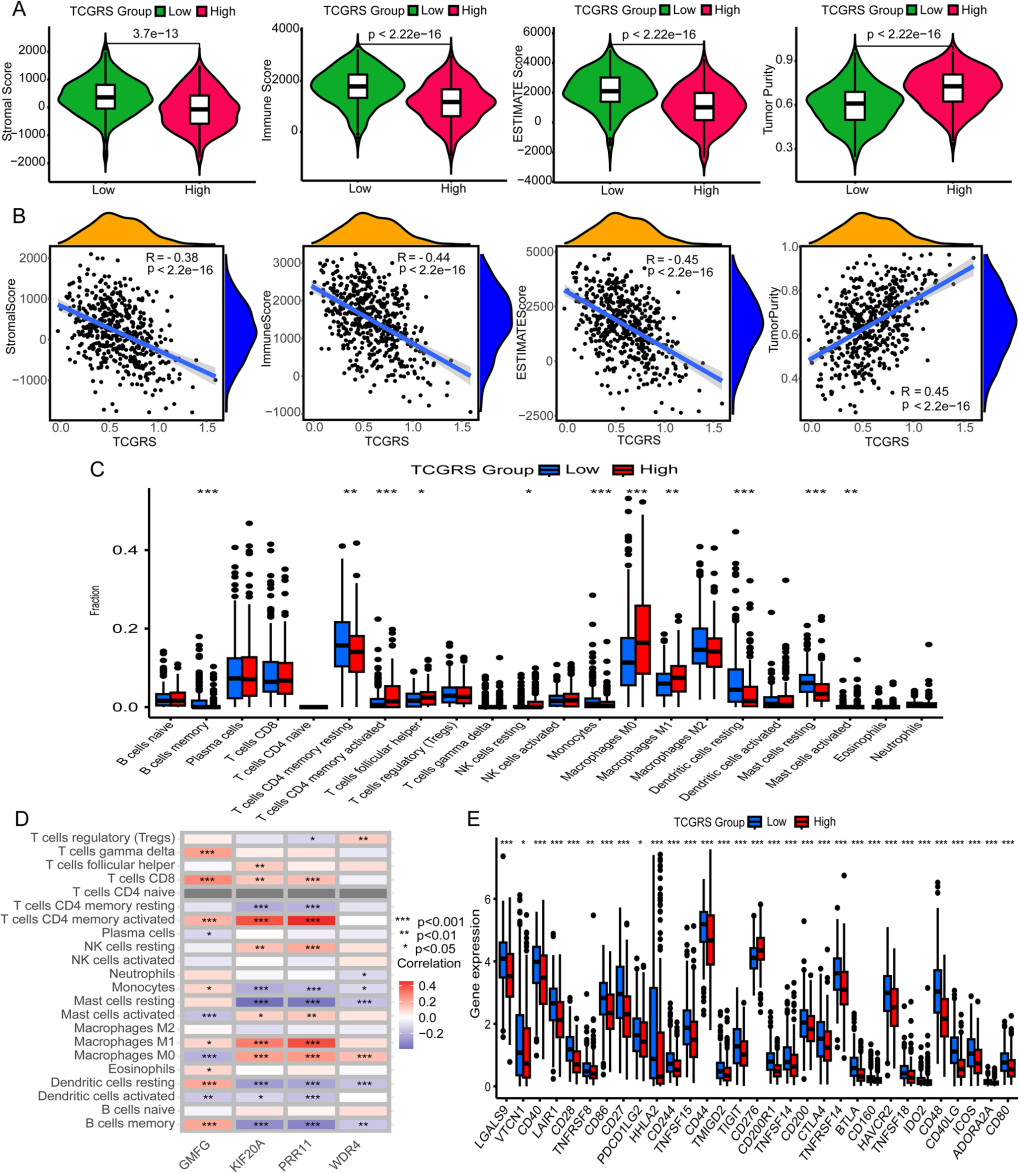
Analysis of TCGRS and immune-related signatures. **A** Analysis of differences in the stromal score, immune score, estimate score, and tumor purity between high and low TCGRS groups. **B** Analysis of correlations between TCGRS and stromal score, immune score, estimate score, and tumor purity. **C** Analysis of differential expression of 22 immune cells in high and low TCGRS groups. **D** Analysis of correlations between model genes and 22 immune cells. **E** Differential expression analysis of multiple immune checkpoints in high and low TCGRS groups. * *p* < 0.05, ** *p* < 0.01, *** *p* < 0.001

TME analysis revealed distinct characteristics between the high and low TCGRS groups. The low TCGRS group exhibited higher stromal, immune, and "ESTIMATE" scores, indicating the increased number of stromal and immune cells within the TME. Conversely, the high TCGRS group showed higher tumor purity (Fig. 13A). Correlation analysis demonstrated a negative association between TCGRS and stromal, immune, and "ESTIMATE" scores, and a positive correlation was observed with tumor purity (Fig. 13B). Furthermore, immune infiltration analysis revealed contrasting profiles between the two groups. The high- TCGRS group exhibited elevated levels of activated CD4 memory T cells, T-cell follicular helper cells, resting natural killer (NK) cells, and M0 and M1 macrophages. In contrast, the low- TCGRS group exhibited increased levels of memory B cells, resting CD4 memory T cells, monocytes, resting dendritic cells, and resting and activated mast cells (Fig. 13C). The correlation between model genes and immune cell populations is depicted in Fig. 13D. Moreover, an examination of immune checkpoints revealed differential expression of 31 out of 47 known immune checkpoints between the high and low TCGRS groups. CD276 exhibited higher expression levels in the high- TCGRS group, while other immune checkpoints showed higher expression levels in the low TCGRS group.

### Immunotherapy prediction and drug sensitivity analysis

**Fig. 14:**
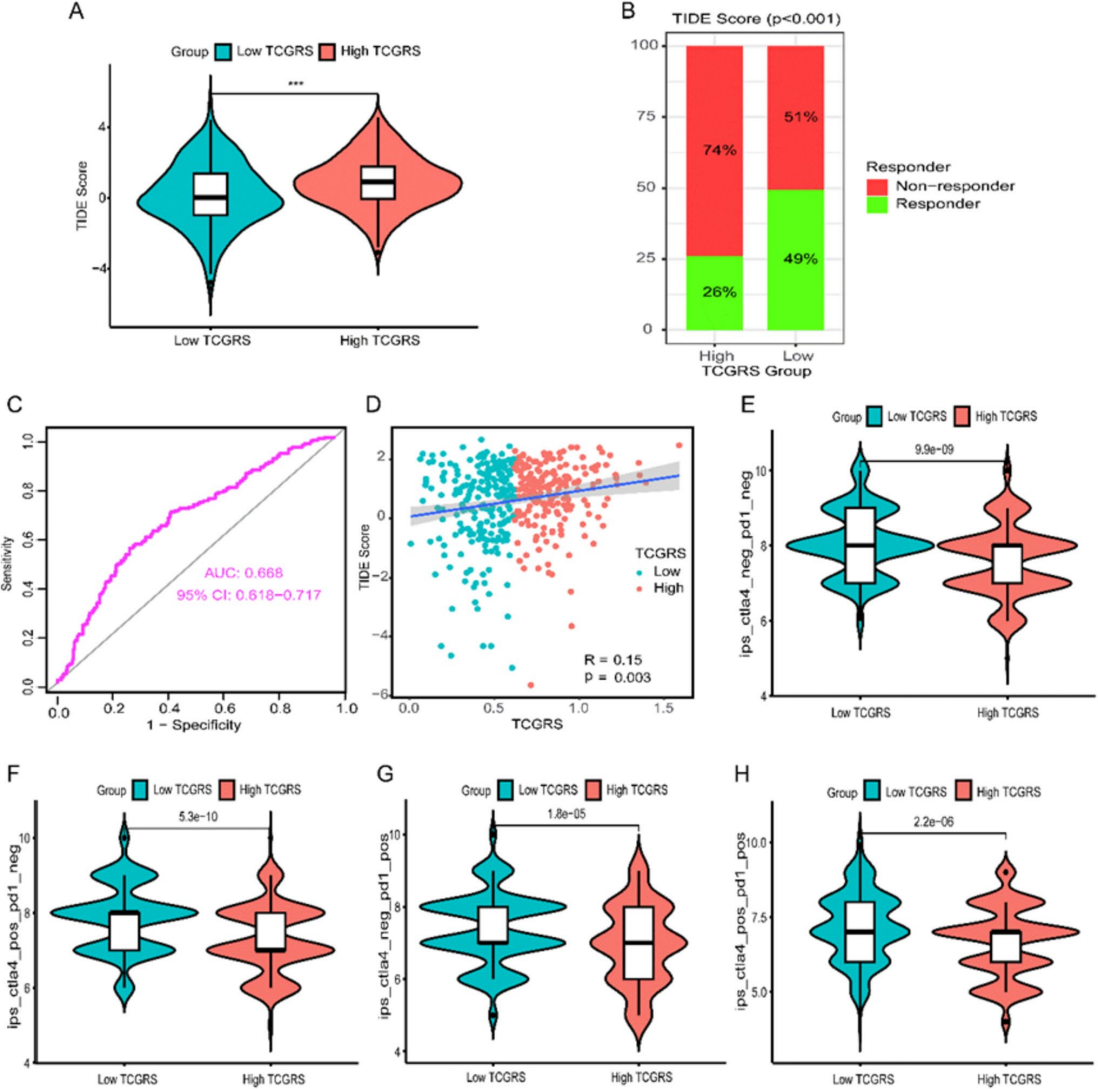
Predicting the effectiveness of immunotherapy based on calculated TCGRS from the TCGA-LUAD cohort model. **A** Analysis of differences in TIDE scores between high and low TCGRS groups. **B** Comparison of response and non-response rates between high- and low- TCGRS groups **C** The ROC curves reflect the accuracy of the TCGRS in predicting immunotherapy. **D** Analysis of correlations between TCGRS and TIDE scores. **E** Analysis of differences in “ips_ctla4_neg_pd1_neg” between high- and low TCGRS groups. **F** Analysis of differences in “ips_ctla4_pos_pd1_neg” between high- and low TCGRS groups. **G** Analysis of differences in “ips_ctla4_neg_pd1_pos” between high- and low TCGRS groups. **H** Analysis of differences in “ips_ctla4_pos_pd1_pos” between high- and low TCGRS groups. *** *p* < 0.001

**Fig. 15:**
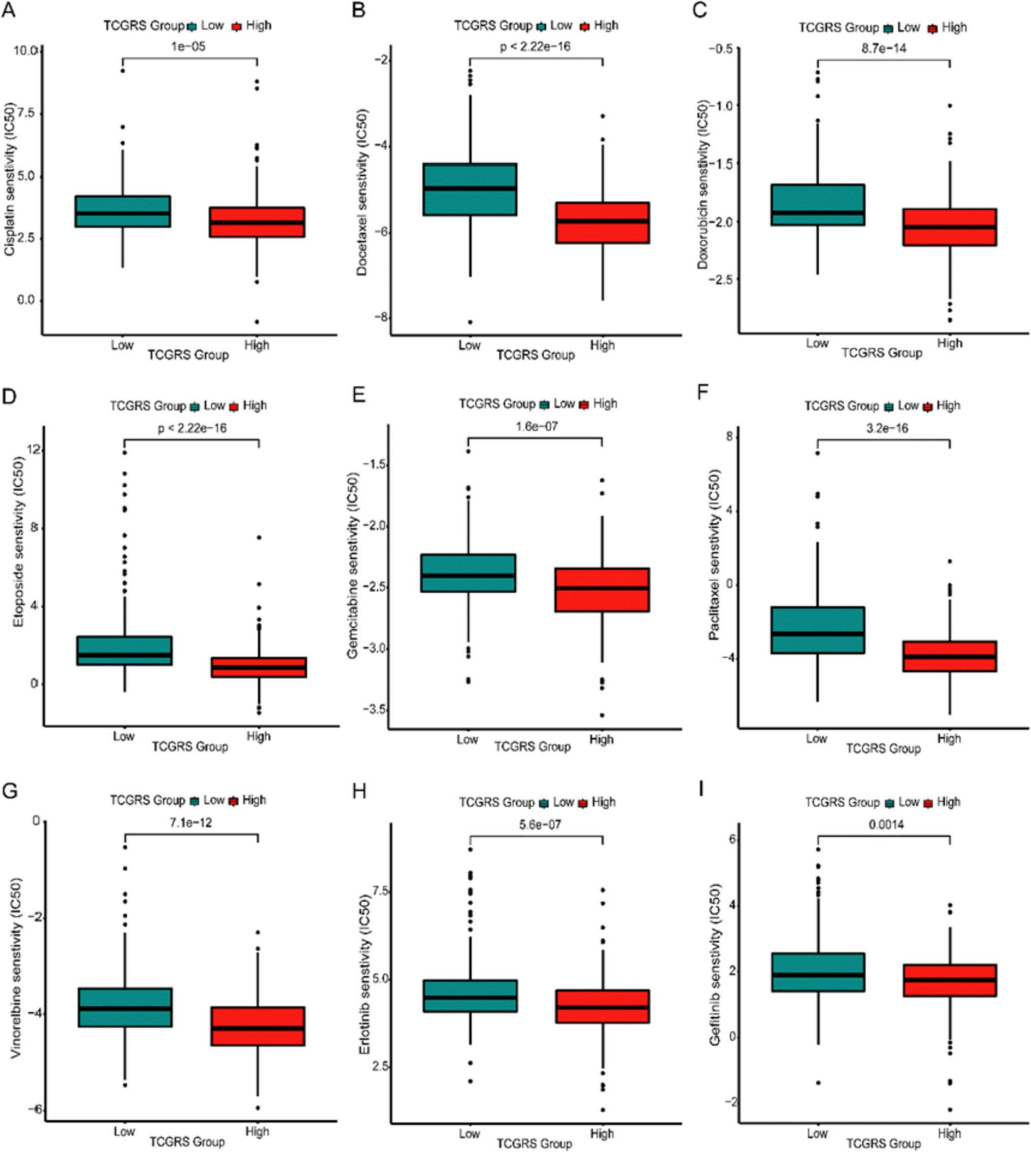
Drug sensitivity analysis of high- and low TCGRS groups in different chemotherapeutic and molecularly targeted drugs. **A** cisplatin. **B** docetaxel. **C** doxorubicin. **D** etoposide. **E** gemcitabine. **F** paclitaxel. **G** vinorelbine. **H** erlotinib. **I** gefitinib

The analysis of immune therapy prediction indicated that the high TCGRS group exhibited considerably higher TIDE scores in comparison to the low TCGRS group (*p* < 0.001) (Fig. 14A). Additionally, the non-response rate was remarkably higher in the high TCGRS group as opposed to the low TCGRS group (74% vs. 51%, *p* < 0.001) (Fig. 14B). The ROC curve demonstrated the favorable predictions capability of TCGRS for immune therapy (Fig. 14C), and a positive association was observed between TCGRS and TIDE scores (Fig. 14D). Moreover, IPS analysis showed that the low TCGRS group exhibited higher scores for “ips_ctla4_neg_pd1_neg”, “ips_ctla4_pos_pd1_neg”, “ips_ctla4_neg_pd1_pos”, and “ips_ctla4_pos_pd1_pos” compared to high TCGRS group (all *p* < 0.001) (Figs. 14E–H). Furthermore, the analysis of drug sensitivity analysis demonstrated that the low TCGRS group demonstrated increased IC50 values for cisplatin, docetaxel, doxorubicin, etoposide, gemcitabine, paclitaxel, vincristine, erlotinib, and gefitinib in comparison to the high TCGRS group (all *p* < 0.01). This indicates lower drug sensitivity in the low- TCGRS group for these specific drugs (Fig. 15).

### Single-cell analysis and protein expression

**Fig. 16:**
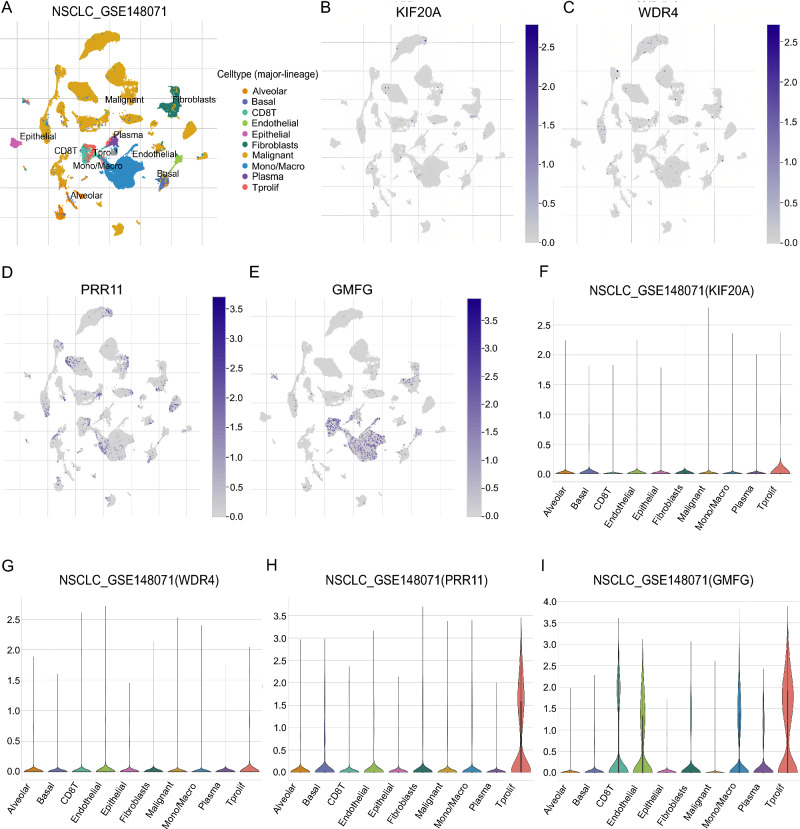
Single-cell analysis of model genes to characterize the microenvironment of the tumor immune system. **A** A UMAP plot is presented to display the ten significant cell populations found in the TME of NSCLC using the GSE148071 dataset. **B** Distribution of KIF20A in cell populations. **C** Distribution of WDR4 in cell populations. **D** Distribution of PRR11 in cell populations. **E** Distribution of GMFG in cell populations. **F** Expression of KIF20A in 10 cell types. **G** Expression of WDR4 in 10 cell types. **H** Expression of PRR11 in 10 cell types. **I** Expression of GMFG in 10 cell types

**Fig. 17:**
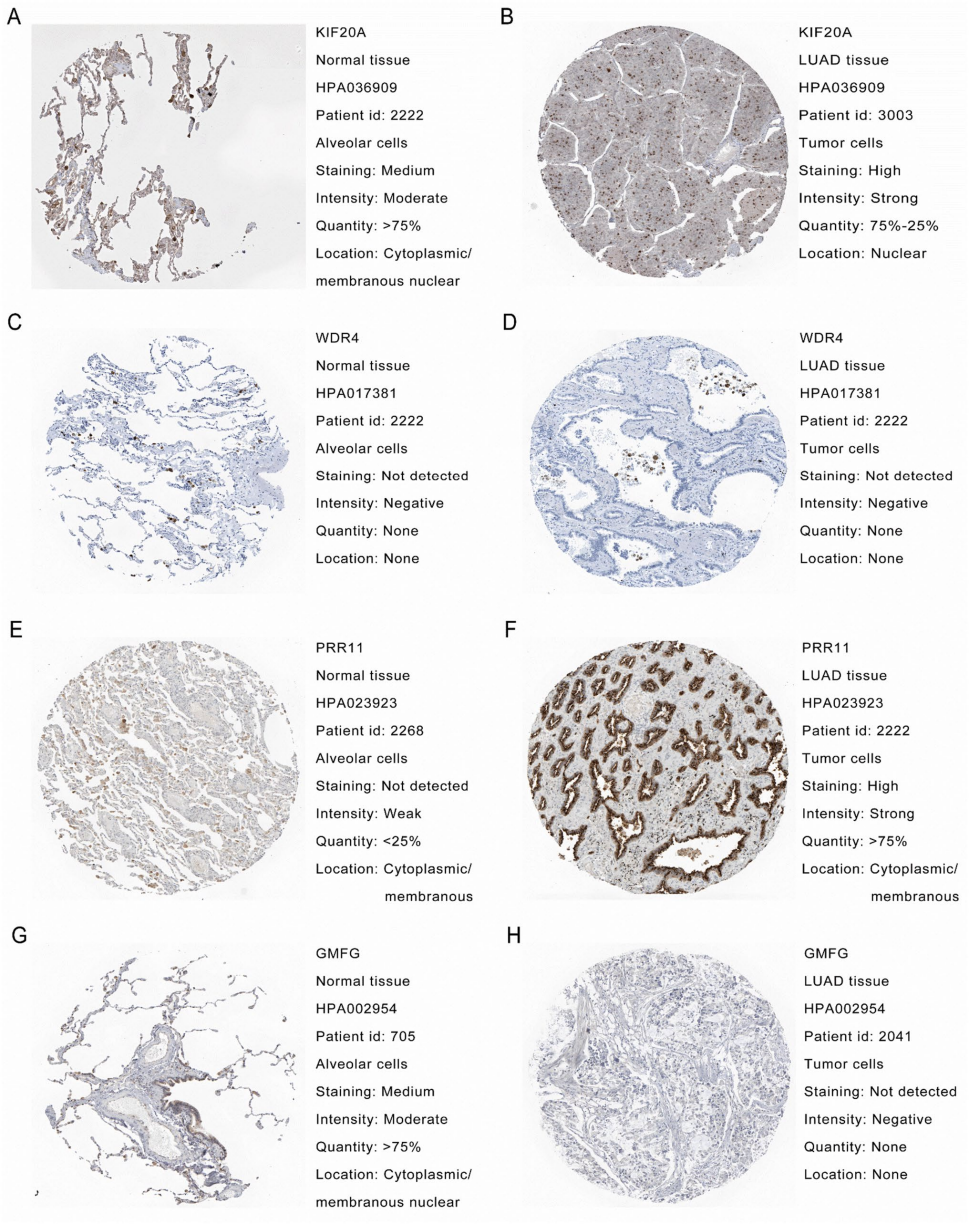
Protein expression levels of model genes were analyzed from the HPA database. **A** Protein expression levels of KIF20A in normal lung tissue. **B** Protein expression levels of KIF20A in LUAD tissue. **C** Protein expression levels of WDR4 in normal lung tissue. **D** Protein expression levels of WDR4 in LUAD tissue. **E** Protein expression levels of PRR11 in normal lung tissue. **F** Protein expression levels of PRR11 in LUAD tissue. **G** Protein expression levels of GMFG in normal lung tissue. **H** Protein expression levels of GMFG in LUAD tissue

Based on GSE148071, single-cell data analysis showed that four model genes were expressed across multiple immune cell types (Fig. 16). Protein expression analysis using the HPA database showed increased expression levels of KIF20A and PRR11 in LUAD tissues. Conversely, GMFG exhibited lower expression levels in LUAD tissues. However, LUAD tissue and normal tissue showed no difference in WDR4 (Fig. 17).

## Discussion

In this investigation, public databases were searched to identify genes that exhibit co-expression with TDP-43 in TCGA-LUAD. Subsequently, *KIF20A*, *WDR4*, *PRR11*, and *GMFG* were chosen based on LASSO regression analysis to construct a risk-scoring model known as the TCGRS model. The TCGRS model demonstrates a high- level of accuracy in predicting the prognosis of LUAD patients. Furthermore, GSEA analysis uncovered a potential association between the high- TCGRS group and various cellular processes, such as the cell cycle, DNA replication, and TP53-related functions and pathways, suggesting a potential influence on tumor stemness and TMB characteristics. The low TCGRS group may be associated with immune-related processes and ways, indicating a more important role for immune cells in this group. According to the examination of TME and immune checkpoints, individuals in the low TCGRS group showed more significant levels of immune cell infiltration and immune checkpoint molecule expression levels in the TME than those in the high TCGRS group. Moreover, the prediction of immune therapy outcomes indicated that the patients in the low TCGRS group displayed lower TIDE scores and more favorable IPS analysis results, suggesting an attenuated immune evasion capacity and a potential for enhanced response to immunotherapy. Analysis of cisplatin, docetaxel, doxorubicin, etoposide, gemcitabine, paclitaxel, vincristine, erlotinib, and gefitinib showed that individuals in the high TCGRS group showed lower IC50 values as compared to those in the low TCGRS group, indicating greater sensitivity to these drug treatments. The high sensitivity of the high TCGRS group to chemotherapy drugs may be due to their involvement in DNA replication- and cell cycle-related pathogenic pathways, as these pathways are the primary targets of most chemotherapy drugs. The expression of four genes was further confirmed at the single-cell level across various immune cell types, highlighting their potential link to immune characteristics. Among these genes, the protein expression levels of KIF20A, PRR11, and GMFG were consistent with their corresponding mRNA expression levels. However, in the case of WDR4, the protein expression did not align with its mRNA expression level, and further investigation is needed on the protein expression of WDR4. This analysis showed that a model developed using genes co-expressed with TDP-43 could predict the prognosis of LUAD in an accurate manner and help select the appropriate treatment for LUAD patients.

This study is the first to use bioinformatics methods to reveal the role of TDP-43 co-expressed genes in LUAD. Investigating these genes is crucial for understanding the biological significance of TDP-43 in tumors. The Kinesin family, discovered in 1985, comprises 14 superfamilies, including kinesin-1 to kinesin-14 (Vale et al. 1985). KIF20A, a member of Kinesin family member 20A, also called Mitotic Kinesin-Like Protein 2 (MKLP2) and RAB6 Interacting, Kinesin-Like (Rabkinesin6) (RAB6KIFL), is located on chromosome 5q31.2 and belongs to Kinesin superfamily-6 (Echard et al. 1998). Members of this gene superfamily play crucial roles in various cellular processes like intracellular transport, spindle assembly, and mitosis (Zhang et al. 2019). Through analysis of public databases and subsequent in vitro and in vivo validation, it has been demonstrated that KIF20A expression is upregulated in LUAD tissues compared to normal tissues. KIF20A is believed to modulate cell proliferation and apoptosis by influencing the cell cycle, thereby producing a malignant phenotype on LUAD (Zhao et al. 2018). WDR4, in conjunction with methyltransferase-like 1 (METTL1), forms a methyltransferase complex responsible for catalyzing the N7-methylguanosine (m7G) modification in eukaryotic transfer RNAs (tRNAs) (Alexandrov et al. 2002; Lin et al. 2018). Studies have shown that METTL1/WDR4 exhibits high expression levels in lung cancer tissues and that the loss of m7G tRNA modification impairs cell proliferation, colony formation, and cell invasiveness, ultimately reducing the tumorigenic potential of cancer cells both in vitro and in vivo (Ma et al. 2021b). Additionally, enhanced expression of METTL1 and WDR4 has been linked to enhanced sensitivity to certain chemotherapy drugs (Duan et al. 2023), potentially explaining the observed chemotherapy sensitivity in the high TCGRS group. *PRR11*, a proline-rich protein-coding gene on chromosome 17q22-23, consists of ten exons and nine introns (Lee et al. 2020; Ji et al. 2013). It participates in various cellular processes of NSCLC cells, including proliferation, migration, cell cycle progression, invasion, apoptosis, and autophagy (Ji et al. 2013; Zhang et al. 2018). Consequently, PRR11 represents a potential therapeutic target for lung cancer treatment. *GMFG*, a gene located on chromosome 19q13.2, comprises seven exons (Tang et al. 2022). It belongs to the actin depolymerizing factor (ADF)/cofilin family, which is crucial in remodeling the actin cytoskeleton (Tang et al. 2022). A previous study suggests that GMFG expression is downregulated in LUAD tissues compared to normal lung tissues and that it may exert anti-cancer effects by activating the p53 signaling pathway, thereby inhibiting the progression of lung cancer (Tang et al. 2022). Furthermore, GMFG has been implicated in immune responses across various cancer types, exhibiting a positive association with immune regulation (Lan et al. 2021). Therefore, the differential biological functions and pathways observed based on GMFG expression levels may offer new insights for personalized treatment approaches in LUAD patients.

Epidemiological studies suggest a close relationship between tumors and neurodegenerative diseases. However, the molecular mechanisms of this relationship still need to be better understood (Campos-Melo et al. 2014). RNA-binding protein (RBP), which exerts a key function in RNA metabolism by participating in the formation of ribonucleic acids, is considered a molecular bridge between these two diseases (Campos-Melo et al. 2014). TDP-43, a multifunctional RNA/DNA-binding protein, plays essential roles in neuronal survival, cell cycle progression, and apoptosis regulation (Han et al. 2013). Its role in tumors has made it a current research hotspot. In NK cells, TDP-43 is a binding target of YTH N6-Methyladenosine RNA Binding Protein F2 (YTHDF2), a member of the YTH domain-containing family. It regulates cell proliferation and survival, thereby influencing the function of NK cells in tumorigenesis (Ma et al. 2021c). TDP-43 might have a role in controlling immunological features in the TME, given that NK cells are a kind of immune cell. In breast cancer, TDP-43 modulates the majority of splicing events via the serine/arginine-rich splicing factor 3 (SRSF3), thereby regulating the progression of triple-negative breast cancer (Ke et al. 2018). Another study demonstrated that TDP-43 might enhance the stemness of breast cancer stem cells through alternative splicing of the CD44 variant (CD44v) (Guo et al. 2022). TDP-43 has been identified in melanoma as a novel oncogene that may regulate tumor growth and metastasis by modulating glucose metabolism (Zeng et al. 2017). In soft tissue sarcomas, bioinformatics analyses have revealed TDP-43 as an essential cancer-promoting gene and its association with prognosis (Wu et al. 2021b). The role of TDP-43 in hepatocellular carcinoma is significant as it regulates glycolysis in cancer cells through transcriptional repression, ultimately leading to a poor prognosis for patients (Park et al. 2013). TDP-43 may regulate tumor progression in cervical cancer through necroptosis-related pathways, with high expression levels indicating a favorable prognosis (Zhan et al. 2022). In conclusion, the relationship between TDP-43 expression and tumor progression has been established (Campos-Melo et al. 2014). However, the exact role of TDP-43 in lung cancer, specifically in LUAD, is not well understood. Exploring the genes co-expressed with TDP-43 could provide valuable insights into its underlying mechanisms in LUAD, as these genes may participate in similar biological functions and pathways. At present, there are no reports in the literature on this topic. Therefore, it is imperative to investigate the genes co-expressed with TDP-43 to enhance understanding of the role played by TDP-43 in LUAD.

This study has several limitations. Firstly, utilizing data from the TCGA and GEO databases may introduce inherent biases associated with data selection. Secondly, to enhance the comprehensive evaluation of the clinical significance of the risk model, it would be beneficial to incorporate additional clinical information and pathological characteristics into the analysis. Additionally, as all data included were obtained from public databases, this study is retrospective; using prospective research methods for survival assessment and treatment prediction would yield more accurate conclusions. Furthermore, the findings of this investigation need to be further verified by more datasets, cell lines, and tissue samples. Lastly, due to the lack of an immunotherapy cohort for LUAD patients, it is not possible to further evaluate the benefits of the constructed model in immunotherapy. Exploring the regulatory mechanisms of immune cell function in the TME has important clinical significance. Moreover, this study has yet to fully elucidate the biological function of TDP-43 co-expressed genes in LUAD, so further exploration is still needed.

## Conclusion

The findings of this study demonstrate the effectiveness of the TDP-43 co-expressed gene model in accurately predicting the prognosis of LUAD individuals while shedding light on the stemness characteristics, TMB, TME, and treatment response of distinct risk groups. The calculated risk score holds promise as a potential biomarker for survival prediction in patients with LUAD, enabling the development of personalized therapeutic approaches. Notably, this study pioneers the construction of a LUAD risk model based on genes co-expressed with TDP-43. These findings provide a theoretical basis for a deeper understanding of the mechanism driving TDP-43 in LUAD, providing opportunities for future developments in novel therapeutic approaches for this disease. By detecting gene expression and calculating risk scores, monitoring these biomarkers in clinical practice can enable the assessment of survival risk and the selecting of appropriate treatment strategies for LUAD patients. This study will likely provide certain assistance in clinical diagnosis and drug development. Additionally, implementing personalized treatment plans based on monitoring these biomarkers can help prevent unnecessary drug-related adverse reactions in patients. This will contribute to patient care and alleviate the economic burden on patients.

## Data Availability

The results of this study can be provided upon request. TCGA-LUAD data was obtained from the TCGA website, while the GSE72094, GSE68465, and GSE41271 datasets were sourced from the GEO database. All data is publicly available and can be used.
